# Contribution of changing precipitation and climatic oscillations in explaining variability of water extents of large reservoirs in Pakistan

**DOI:** 10.1038/s41598-019-54872-x

**Published:** 2019-12-13

**Authors:** Ibrar ul Hassan Akhtar, H. Athar

**Affiliations:** 1 Department of Meteorology, COMSATS University Islamabad, Islamabad, Pakistan; 2 Centre for Climate Research and Development, COMSATS University Islamabad, Islamabad, Pakistan; 3 Space Applications & Research Complex, Pakistan Space and Upper Atmosphere Research Commission, Islamabad, Pakistan

**Keywords:** Governance, Hydrology

## Abstract

Major threat that Pakistan faces today is water scarcity and any significant change in water availability from storage reservoirs coupled with below normal precipitation threatens food security of more than 207 million people. Two major reservoirs of Tarbela and Mangla on Indus and Jhelum rivers are studied. Landsat satellite’s data are used to estimate the water extents of these reservoirs during 1981–2017. A long-term significant decrease of 15–25% decade^−1^ in water extent is found for Tarbela as compared to 37–70% decade^−1^ for Mangla, mainly during March to June. Significant water extents reductions are observed in the range of −23.9 to −53.4 km^2^ (1991–2017) and −63.1 to −52.3 km^2^ (2001–2010 and 2011–2017) for Tarbela and Mangla, respectively. The precipitation amount and areas receiving this precipitation show a significant decreasing trend of −4.68 to −8.40 mm year^−1^ and −358.1 to −309.9 km^2^ year^−1^ for basins of Mangla and Tarbela, respectively. The precipitation and climatic oscillations are playing roles in variability of water extents. The ensuing multiple linear regression models predict water extents with an average error of 13% and 16% for Tarbela and Mangla, respectively.

## Introduction

## The Asian Waters and River Basins

Fresh water resources being scarce can’t be taken as granted and there is a need to identify the hotspots for adaptation in major river basins of Asia^1^. The Indus river basin (IRB) has been identified as most vulnerable to change in water availability due to unpredictable glacier melt and uncertain future precipitation regime^2–4^. The large-scale irrigation systems connected to Indus, Ganges, and Yangtze basins are now facing high irrigation water demand to secure the food for over a billion people through increased crops cultivation and improved yields^5,6^.

Global surface water for irrigation has significantly increased through the construction of large reservoirs during the 20th century^7^. By the end of 20th century, irrigation water supply from reservoirs has increased from 5% to 40% as compared to the start of the 20th century. One of the main reasons is an increase in irrigated areas and building of large reservoirs at global scale. For instance, an evaluation of 1300 reservoirs in California, USA, revealed that 200 reservoirs storage capacity is 45 million acre feet (MAF), which subsides the impacts of extreme climate events^8^. Countries like USA have benefited from economic and societal development through continual construction of dams/reservoirs^9^. Although, major reservoirs always remained a controversial topic, however, changing climate along with an increase in population and higher water demands are leading to question: how well is our assessment of variability in water storage reservoirs? The IRB like other major river basins in Asia^10^, was and is facing most of key challenges related to ongoing climate change events triggered by population growth, environmental degradation, urbanization, poor water governance, and political instability. The IRB system is spread across four countries namely, Pakistan, India, Afghanistan and China^11^. It is semi closed with a foremost opportunity to add value to water through storages^12^.

## Water Situation in Pakistan

The Indus basin irrigation system representing the largest irrigation network of world has only two large storage reservoirs namely, Tarbela on Indus river and Mangla on Jhelum river in Pakistan^5^. Any abrupt change in water supply from these upstream reservoirs coupled with downstream below normal precipitation leads to high losses to Pakistan’s economy and threatens the food security. There is no single large reservoir on Chenab river, mainly due to terrain limitation and lack of water resource planning^13^. Increased population and geo-political landscape are leading towards scarcity in available water resources^14^. Irrigated economy is based on GDP contribution of major crops, livestock and other crops which are under threat of changing climate^15^. Water coming from high mountain areas of IRB is a key element of livelihood as it feeds the Indus basin irrigation system^16^. Water reservoirs are mostly considered as a source of hydro energy^17^. Pakistan has insufficient water storage capacity as compared to other countries like USA, China and India^18^. Water reserves in Tarbela and Mangla support irrigated system spread across Punjab and Sindh for only 20 days as compared to 220 days in India^19,20^. Pakistan’s smaller storage capacity makes it essential to invest in reservoirs, given highly variable river flows and changing climate. It is proposed that an integrated water resource management of IRB be adapted, given that it is a highly climate vulnerable country and being dependent largely on water originating from high mountains^21^.

**Figure 1:**
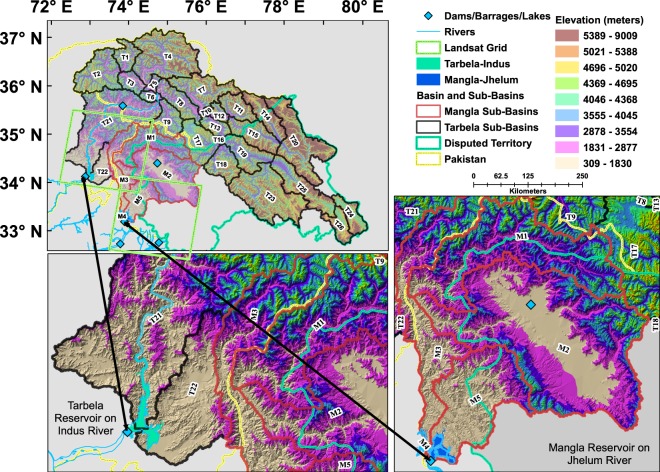
Study area showing the Tarbela-Indus and Mangla-Jhelum reservoirs, associated basins and sub-basins of PUIRB. Tarbela is the only major reservoir in PUIRB stretching from Gilgit in north Karakoram to eastern and central Himalaya in disputed Kashmir and in north western India. Mangla reservoir is spread across Azad and Jammu Kashmir and considerably smaller in term of basin size. The ALOS GDEM based topography shows the diversification of ecosystem that exists in basins of both reservoirs. Two Landsat satellite grids show the image foot prints. Landsat satellite data were obtained from the Earth Resources Observation and Science (EROS) Center of the United States Geological Survey, USA (https://earthexplorer.usgs.gov/) and ALOS GDEM data were obtained from ALOS Science Project, Earth Observation Research Center (EORC), Japan Aerospace Exploration Agency (JAXA) (https://www.eorc.jaxa.jp/ALOS/en/aw3d30/).

A detailed analysis at river basin scale indicates that irrigation reservoirs are able to make more water available in specific seasons only, especially in some Asian basins, where natural water availability is highly variable throughout the year^7^. Two largest reservoirs of Pakistan constitute the sole sources of water availability for irrigation purposes and have operational impacts on various societal sectors such as generation of 60% of hydropower. At the same time, these reservoirs lay foundation for food security for a population of over 207 million^22^. Pakistan is facing rising water scarcity which is leading to socio-economic impacts on various developmental sector’s growth^23^. Tarbela and Mangla reservoirs are multi-purpose storage reservoirs and supply irrigation water to 18.2 million hectares with a distribution of 84% for Kharif/summer and 16% for Rabi/winter crops^24,25^. Part of the upper Indus river basin (PUIRB) of two reservoirs covers around 0.171 million square kilometer (km^2^), and is spread across Gilgit Baltistan, Khyber Pakhtunkhwa, Azad and Jammu Kashmir in Pakistan and in disputed occupied Kashmir in India (Fig. 1 and Extended Table 1).

The hydrology and water management department of Pakistan, referred to as water and power development authority (WAPDA) provides information related to water reserves in the two large reservoirs. Water reserves are around 9.87 MAF in August, 2019 as compared to 6.43 MAF in August, 2018 which is still lower than last five years (2014–2018) average of 10.6 MAF during August and is associated with a big gap in storage capacity and inflows^26^. This is due to lower than normal precipitation in the basin areas triggered by climate change during 2018^27^. The situation can be alleviated through only exceptional monsoon or early glacial melting. Thus, a detailed study incorporating these recently changing precipitation patterns is timely for assessment of their influence on the two large water reservoirs of Pakistan.

## Climatic Variability and Water Reservoirs in Pakistan

The large reservoirs are considered as an integral part of the surface water hydrology through their influential role in diluting the extreme events of floods and drought along with optimum water resource management at regional scale^28^. These large reservoirs have undoubtedly improved the socio-economics of the countries by improving the economic growth as well as by alleviating poverty^29^. Large reservoirs have both positive impacts by providing the water during deficit periods and negative impacts by changing the local basin environment through reservoir effects^30^. A recent study has demonstrated that how precipitation and El Niño Southern Oscillations or ENSO can be linked with remote sensing data-based land covers through vegetation proxies in Indonesia^31^. In irrigated areas, vegetation sensitivity is more linked with water availability through storage reservoirs. We attempted to link remote sensing-based water extents (WEs) of the two reservoirs with gridded precipitation and climatic oscillations (COs) of ENSO, North Atlantic Oscillations or NAO and Indian Ocean Dipole oscillations or IOD in this study.

**Figure 2:**
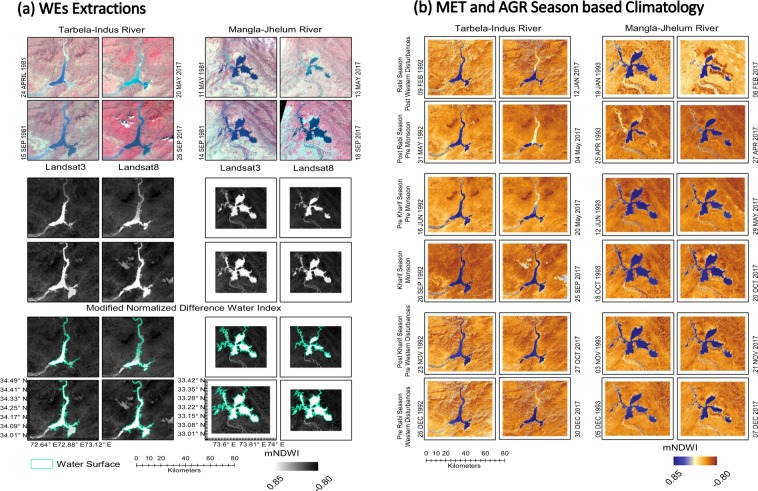
Optical Landsat satellites series data (3, 5, 7 and 8) are used to extract the WEs for Tarbela and Mangla reservoirs covering 37 years (1981–2017). (**a**) Shows extracted WEs from multispectral Landsat imagery based on NDWI and conversion to vector layer. (**b**) MET and AGR seasons based temporal evolution of WEs for Tarbela (1992–2017) and for Mangla (1993–2017). Four out of six seasons show major reduction in WEs for Tarbela & Mangla starting from Pre Rabi to Pre Kharif (WDs to Pre MS) covering December to June. Landsat satellite data were downloaded from the Earth Resources Observation and Science (EROS) Center of the United States Geological Survey, USA (https://earthexplorer.usgs.gov/).

Thus, an updated view of the monthly, seasonal and inter-annual variability of WEs of large reservoirs along with role of upstream precipitation, precipitation surface area and COs is provided. This will deliver the basic information about WE of reservoirs which is considered as an equivalent to water storage volume^32–34^. This will also help the policy makers for informed decisions related to future water apportionment and allocations under normal, flood, and drought conditions and ensure more objectivity in water sharing among various stakeholders^35,36^. Long term (1981–2017) Landsat satellites data are analyzed to optically estimate the WEs of the Tarbela reservoir in Khyber Pakhtunkhwa (34.09°N & 72.70°E) and Mangla reservoir in Azad and Jammu Kashmir (33.15°N & 73.64°E), for the first time (Fig. 2). This is to explore the co-variations between WEs and precipitation amount, surface area receiving this precipitation at basin and sub**-**basin scale, and their possible linkages with global COs (ENSO, NAO and IOD).

## Results

### Remote sensing and WE variations

**Figure 3:**
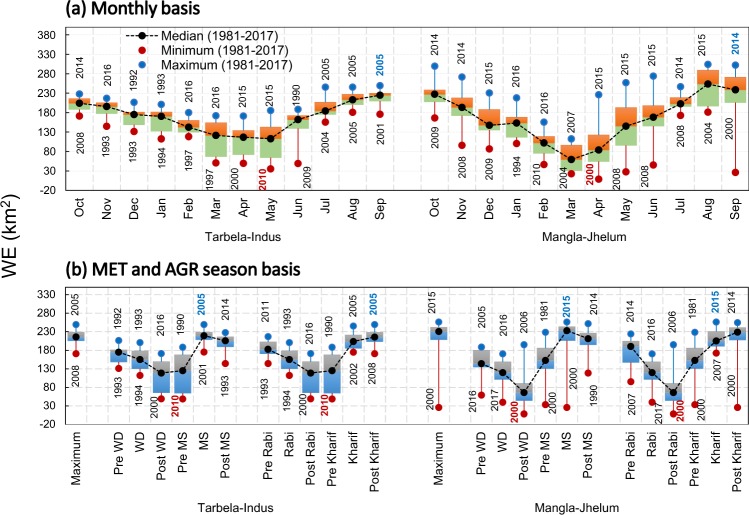
The WEs of Tarbela and Mangla reservoirs at (**a**) monthly and (**b**) seasonal scale during 1981–2017. (**a**) Monthly WEs show less variable curve representing the water storage in Tarbela as compared to Mangla during 1981–2017. This is mainly attributed to hydro-meteorological regimes in the respective basins. Driest/Wettest months recorded are May, 2010/ Sep, 2005 and Apr, 2000/ Sep, 2014 for Tarbela and Mangla, respectively. (**b**) The MET and AGR seasons also showed distinct WE behavior for both reservoirs.

Monthly and seasonal variability in WEs are presented for the study period of thirty-seven years (Fig. 3). Monthly WE variability shows distinctive temporal profile for both reservoirs. Tarbela shows less steep decrease and increase in WEs as compared to Mangla. October represents the first month after the end of Monsoon (MS) season in PUIRB and start of Rabi cropping season in downstream Punjab. These WE variations have increased in last 15 to 25 years and reached to dead storage level during 2000 and 2010 for Mangla and Tarbela, respectively.

Mangla WE reaches its dead level by March of each year and ranged between 22 km^2^ (2004) and 112 km^2^ (2007). This is due to the fact that basin area of the Mangla is smaller, less glaciated, and monsoon precipitation is a major source of water accumulation. Tarbela reservoir reaches its dead water level by the end of May each year and ranged between 35 km^2^ (2010) and 185 km^2^ (2015). This relatively prolong time to reach the dead level in Tarbela is due to the large basin area as well as more glaciated areas contributing to water inflows to reservoir along with precipitation received from Western Disturbances (WDs) as compared to Mangla.

Both reservoirs have seasonality in their WEs. Mangla reservoir reaches its maximum WE by August and ranged between 181 km^2^ (2004) and 304 km^2^ (2015). Tarbela reservoir reaches its maximum by end of September each year and ranged between 175 km^2^ (2001) and 249 km^2^ (2005). In comparison, worst/best years in term of WEs are observed to be May, 2010/September, 2005 and April, 2000/September, 2014 for Tarbela/Mangla reservoir, respectively. Seasonal maximum WEs of 171/249 km^2^ are observed during 2008 and 2005 for Tarbela in comparison to 26/256 km^2^ for Mangla.

Tarbela reservoir showed maximum variations in WEs during Post WD/Post Rabi and Pre MS/Pre Kharif seasons ranging from 49 km^2^ to 188 km^2^. Mangla showed variations throughout six seasons. Tarbela and Mangla showed highest/lowest WEs during MS and Post Kharif of 2005/Pre MS and Pre Kharif of 2010 and MS and Kharif of 2015/Post WD and Post Rabi of 2000. These variations are considered as a major influencing factor in terms of water availability for downstream crops production. We explored these WE variations of the reservoirs by linking them with upstream precipitation regimes, and with global COs.

### Precipitation and WE trends

**Figure 4:**
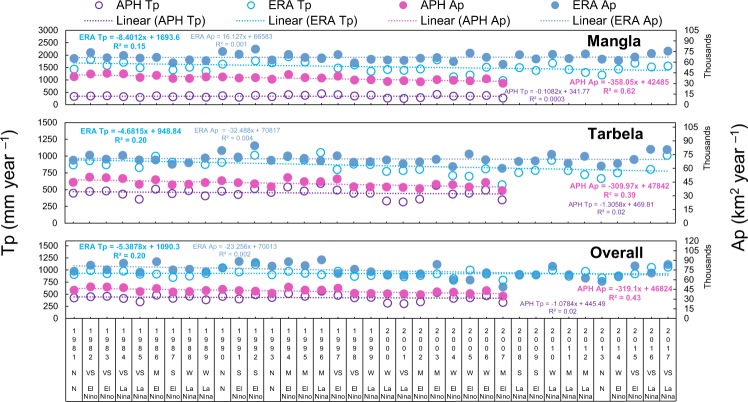
ERA and APH data based linear trends for Mangla-Jhelum basin, Tarbela-Indus basin and overall basin. The ERA based Tp and APH based Ap show a significant decrease of 5.38 mm year^−1^, 4.68 mm year^−1^, 8.40 mm year^−1^ and 319 km^2^ year^−1^, 310 km^2^ year^−1^ and 358 km^2^ year^−1^, respectively. Two very strong El Niño events of 1982–1983 and 1997–1998 reduced Tp (from 481 mm year^−1^ to 292 mm year^−1^ and 591 mm year^−1^ to 446 mm year^−1^ for 1982–1983 and 1997–1998). A similar decrease in Tp has been observed for Mangla-Jhelum basin during 1982–1983 and 1997–1998 (from 358 mm year^−1^ to 292 mm year^−1^ and 438 mm year^−1^ to 346 mm year^−1^, respectively). The Ap also decreased from 50233 km^2^ year^−1^ to 42442 km^2^ year^−1^ and 48286 km^2^ year^−1^ to 39758 km^2^ year^−1^ for Tarbela during 1982–1983, and 1997–1998. Mangla also shows a decrease in Ap during 1982–1983, 1997–1998 and 2007 (44180 km^2^ year^−1^ to 38432 km^2^ year^−1^, 40199 km^2^ year^−1^ to 34740 km^2^ year^−1^ and 36543 km^2^ year^−1^ to 30230 km^2^ year^−1^, respectively). Strength of ENSO phases are represented by VS (very strong), S (strong), M (moderate), W (weak) and N (neutral).

The European Centre for Medium-Range Weather Forecasts (ECMWF) based ERA Interim data hereafter referred to as ERA and Asian Precipitation Highly-Resolved Observational Data Integration towards Evaluation (APHRODITE) based data hereafter referred to as APH are two independent gridded precipitation datasets at global and regional scales, respectively^10^. Total precipitation amount (Tp) and precipitation receiving surface area (Ap) are computed for Tarbela, Mangla and for overall basin. The ERA Tp and APH Ap showed significant annual linear decreasing trend and is associated with ENSO episodes (Fig. 4). The APH Ap showed significant decreasing trends of −358.1 (R^2^ = 0.62), −309.9 (R^2^ = 0.39) and −319.1 (R^2^ = 0.43) km^2^ year^−1^ for Mangla, Tarbela and overall basin, respectively. The ERA Tp showed significant negative linear trends of −4.68 to −8.40 mm year^−1^ (R^2^ = 0.15 to 0.20). No significant trend is observed for APH Tp and ERA Ap. Categorized annual ENSO episodes suggest that the decreases in Tp and Ap are linked to El Niño and La Niña events. This highlights that there are long term decreasing trends in upstream Tp and Ap linked to COs.

Decadal percent changes are computed in WEs, Tp, and Ap (Extended Fig. 1, Extended Table 2). Tarbela reservoir WE shows a reduction of 15–25% decade^−1^ with reference to normal based on 1981–2017. Maximum negative change has occurred during Post WD (Post Rabi) and Pre MS (Pre Kharif) with more than 50% change decade^−1^. The APH Tp and APH Ap are found more variable during Pre WD (Pre Rabi) while during rest of seasons, the change is around ±10% decade^−1^. The ERA Tp shows a positive change of 15 to 20% decade^−1^ during 1981–2000. Least decadal changes are observed for the ERA Tp. The Mangla reservoir showed even more seasonal fluctuations in WE at decadal scale. This WE has suffered highest decline of more than 75% decade^−1^ during Post WD, Pre MS, MS and Post Kharif seasons of 1991–2000. These changes have been observed in the range of 37% to 70% change decade^−1^ during 2001–2017. Similar variation patterns are observed in ERA and APH Tp for Mangla reservoir albeit with higher amplitudes.

Significant decadal changes are observed for WEs in the range of −23.9 to −53.4 km^2^ (1991–2017), ERA Ap of −223.8 to −242.2 km^2^ (2001–2010), and APH Ap of 228.9 to 196.1 & −277.6 to −284.7 km^2^ (1981–1990 and 2001–2007) during MET and AGR seasons in Tarbela basin. Mangla basin showed significant decadal change in WEs in the range of −63.1 to −52.3 km^2^ (2001–2010 and 2011–2017) and in APH Ap ranging from 276.0 to 232.9 & −325.4 to −308.9 km^2^ (2001–2007 and 1981–1990) during MET and AGR seasons.

### Relationship with COs and precipitation

**Figure 5:**
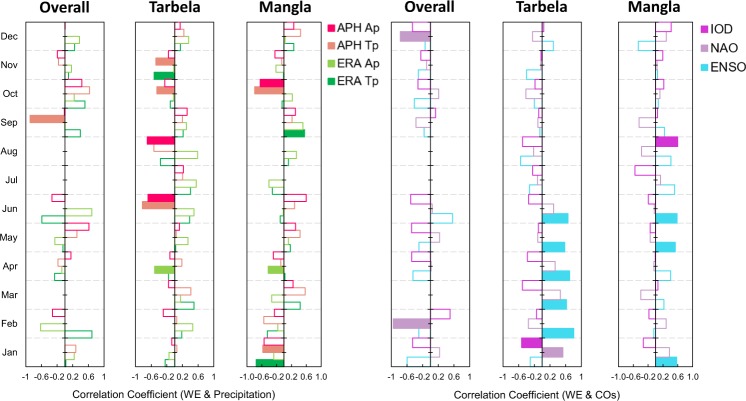
Correlation analysis of WEs with ERA and APH based Tp and Ap & COs. Filled bars show that correlation is significant at 95% confidence level. Overall basin shows least significant and negative correlations with precipitation and COs. The Tarbela reservoir shows negative significant correlations with precipitation from mid to end of the year. However, positive correlations are observed with NAO and ENSO and negative correlations with IOD during winter to early summer. Mangla reservoir shows significant correlations with precipitation but for different months. Significant positive correlations are observed for ENSO and IOD only.

The variations in WEs are correlated with Tp, Ap and Sea Surface Temperature Anomalies (SSTA) for the three different COs (Fig. 5). Mangla WE showed significant negative correlation during January, April and October attributing to less precipitation from WD except for September. Significant positive correlation was found during September owing to strength of the MS ERA Tp. The Tarbela and overall basin revealed significant negative correlations with Tp and Ap at varying monthly timescale. The COs have both positive and negative correlations with Mangla and Tarbela WEs, depending upon the month of the year. Mangla reservoir is significantly sensitive to positive phase of the ENSO and the IOD. Tarbela correlations showed more strong positive relationship with ENSO (January to June) and NAO (January) and negative correlation with the IOD. Overall basin showed strong negative correlation between WE change and the NAO.

Further, COs along with Tp and Ap are correlated at monthly scale to assess their co-variability over the Tarbela, Mangla and overall basins (Extended Fig. 2). Correlation magnitude and sign depends upon the geographical location of the reservoir and time of the year. The ENSO was observed as most influencing the Tp and Ap for Mangla basin followed by Tarbela basin and overall basin than NAO and IOD. The ENSO and Tp over Mangla basin showed the highest co**-**variability. The ENSO showed maximum range of variability in cumulative SSTA (CSSTA) from ≤−9.5 to ≥9.5 °C year^−1^ and least CSSTA is observed for IOD (≤−1.8 to ≥9.5 °C year^−1^). Flood and drought years (provided by Pakistan Meteorological Department) are compared with the identified extreme wet to extreme dry years for ENSO, NAO and IOD oscillations (Extended Fig. 3). Three reported extreme floods of 2010, 2011 and 1992 showed association with extreme wet class for ENSO, NAO and IOD, respectively. The ENSO and IOD is observed to link more with drought events in Pakistan as compared to NAO. In general, south Asia is also showing an increasing trend of flood events from 6 in 1981 to 20 in 2017 as compared to drought events.

Mann Kendall (MK) and Sen’s slope trend analysis has been applied at three month running mean, as well as at seasonal and annual time scale SSTA for COs (Extended Tables 3 and 4). No significant trends are observed in ENSO SSTA at three months and seasonal time scales. The Tarbela, Mangla and overall basin referenced years showed significant decreasing trend of NAO SSTA from −0.03 to >−0.06 °C month^−1^ year^−1^ for MJJ, JJA and JAS of summer season (summer season extends from May to September). Tarbela reference years show no significant trend in IOD based SSTA. The IOD shows significant increasing trend of 0.010 to >0.014 °C month^−1^ year^−1^ for SON, OND and NDJ during winter season (winter season extends from November to March). There is a significant increase in summer as well as in winter IOD SSTA of 0.011 to >0.024 °C month^−1^ year^−1^. The NAO based SSTA displayed significant decreasing trends from 0.020 to >0.243 °C season^−1^ year^−1^, for MS and Kharif seasons. The Tarbela related IOD SSTA is not showing any significant trend, while Mangla is displaying an increasing trend of 0.010 to >0.014 °C season^−1^ year^−1^ in IOD SSTA for Pre WD (Pre Rabi) and Post MS only.

### Sub-basin scale trends analysis

**Figure 6:**
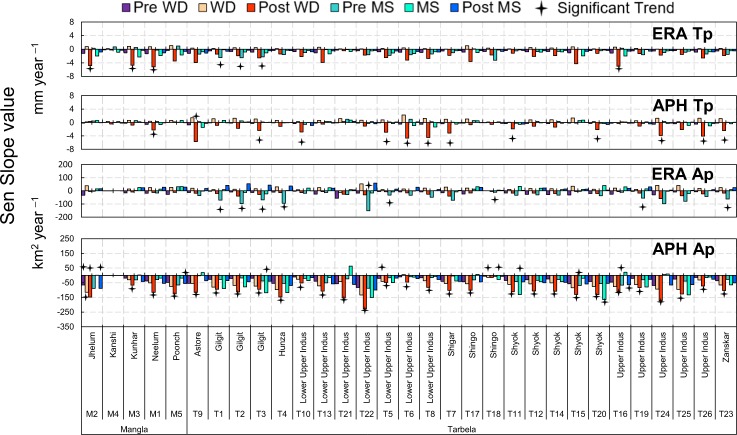
The MK and Sen’s slope trend analysis for ERA and APH based Tp and Ap at sub-basin scales. Bars with four-point star represent the significant trends at 95% confidence level. The ERA Tp and Ap show decreasing trends mostly in Mangla and some of Tarbela sub-basins during post WD and pre MS. Most of the significant decreasing trends are observed for APH Tp and Ap throughout the sub-basins of PUIRB during post WD, pre MS and MS seasons.

Seasonal MK and Sen’s trends analysis is presented for ERA and APH based Tp and Ap at sub**-**basins scale (Fig. 6). The ERA Tp shows a significant decreasing trend of 4.7 to 5.2 mm season^−1^ year^−1^ in Jhelum, Kunhar, Neelum sub**-**basins of Mangla and upper Indus sub**-**basin of Tarbela during Post WD. Gilgit sub**-**basin is displaying a significant decreasing trend of 2.3 to 2.5 mm season^−1^ year^−1^ in ERA Tp during Pre MS season. The APH Tp is displaying significant decreasing trends in Post WD mostly for the sub**-**basins of Tarbela (1.9 to 5.7 mm season^−1^ year^−1^) as compared to Neelum sub**-**basin of Mangla (2.2 mm season^−1^ year^−1^). The ERA Ap suggests a significant decrease of 11 to 152 km^2^ season^−1^ year^−1^ only for Tarbela sub**-**basins of Gilgit, Hunza, lower part of upper Indus, Shingo, Upper Indus and Zanksar. However, APH Ap shows maximum seasonal decreasing trends across sub**-**basins of both Mangla and Tarbela. Jhelum sub**-**basin of Mangla shows a significant seasonal decrease of 114 to 148 km^2^ season^−1^ year^−1^ during Pre WD, WS, Post WD and Post MS. The sub**-**basins of Kunhar, Neelum and Poonch are displaying a decrease in Ap only in Post WD (66 to 126 km^2^ season^−1^ year^−1^). For Tarbela, 25 out of 26 sub**-**basins show significant decreasing trends of 48 to 228 km^2^ season^−1^ year^−1^ during Post WD. The MS precipitation is significantly decreasing only in Gilgit and Shyok sub**-**basins.

### Multiple linear regression models

Multiple linear regression models (MLRs) are developed for the prediction of WEs of the two reservoirs using monthly variables of ENSO, NAO, IOD, ERA and APH Tp & Ap (Table 1). We have addressed the issue of over fitting, high R^2^ value, and spurious relationships for 148 predictor variables (n = 19,388) through steps of principal component analysis (PCA), and multi-collinearity removal (variance inflation factor <2.0). The Tarbela WE can be predicted based on stepwise MLR model using 8 different explanatory variables of ENSO, ERA Tp of T6, T25, GB520P, GL5D, ERA Ap T3, T9 and APH Ap T9 (training data set R^2^ = 0.626; RMSE = 33.01 km^2^ and validation data set with same R^2^ and RMSE = 69.92 km^2^). The ERA Tp of disputed Kashmir sub**-**basin of T25 is observed as the most influential variable. Best model approach based on best adjusted R^2^ is employed to identify best MLR model for Mangla reservoir. The WE of Mangla can be predicted by using 10 variables of NAO, ERA Tp M1, M2, APH TpGL5, GL5D, APH Ap M4, M5, GL5, GL5P, GL5D (training data set R^2^ = 0.509; RMSE = 48.16 km^2^ and validation data set with same R^2^ and RMSE = 92.47 km^2^). The APH Ap in GL5P sub**-**basin is observed as the most influential variable.

**Table 1:** Multiple linear regression models are developed to predict the WEs of the Tarbela and Mangla reservoirs.

Reservoirs	Predictors	Numbers of variables	Fitting Model	Criteria	MLR Models							
					ANOVA			RMSE (km^2^)		Model Equation	Number of Significant Predictors	Most Influential Predictor
					R²	Adj R²	df	Training	Validation			
Tarbela	Years, ENSO, NAO, IOD, ERA Tp and ERA Ap	78	Best Model **Stepwise** Forward	Best Adjusted R² In: P value 0.05 Out: P Value 0.10 P value 0.05	0.56 **0.59** 0.52	0.53 0.57 0.49	61 63 66	35.0 **33.5** 35.9	72.9 122.6 60.2	WE = 159.9-0.007×APH Ap-2.57×ERA Tp GB520P+2.48×ERA Tp GL5P-0.039×ERA Ap GL5+0.023×ERA Ap GG20P+0.009×ERA Ap GB520P+0.036×ERA Ap GL5P-0.027×ERA Ap GG20D-0.037×ERA Ap GB520D+0.035×ERA Ap GL5D WE = 27.4-0.006×APH Ap+0.64×ERA Tp T17+0.74×ERA Tp T21+0.027×ERA Ap T3-0.03×ERA Ap T15+0.027×ERA Ap T17 WE = 160.1-0.66×ERA Tp T15+0.94×ERA Tp T17-0.79×ERA Tp T25+0.043×ERA Ap T3-0.007×ERA Ap GB520P+0.0005×ERA Ap GG20D	10 6 6	ERA Tp GB520P **ERA Ap T3** ERA Ap T3
	Years, ENSO, NAO, IOD, ERA Tp, ERA Ap, APH Tp and APH Ap	148	Best Model **Stepwise** Forward	Best Adjusted R² In: P value 0.05 Out: P Value 0.10 P value 0.05	0.52 **0.63** 0.56	0.45 0.58 0.53	120 124 124	38.4 **33.0** 36.4	108.1 69.9 61.1	WE = 207.3-4.46×ERA Tp GB520P+4.42×ERA Tp GL5P+0.16×APH Ap T26-0.032×APH Ap GL5-0.017×APH Ap GG20P-0.047×APH Ap GB520P+0.03×APH Ap GL5P+0.008×APH Ap GG20D+0.07×APH Ap GB520D+0.041×Aph Ap GL5D WE = 161.5+12.57×ENSO+1.08×ERA Tp T6-3.27×ERA Tp T25-2.73×ERA Tp GB520P+4.12×ERA Tp GL5D+0.035×ERA Ap T3-0.022×ERA Ap T9-0.012×APH Ap T9 WE = 171.5+16.72×ENSO-0.93×ERA Tp T25+0.016×ERA Ap T3-0.02×ERA Ap GG20D+0.0075×ERA Ap GL5D	10 8 5	ERA Tp GB520P **ERA Tp T25** ERA Tp T25
Mangla	Years, ENSO, NAO, IOD, ERA Tp and ERA Ap	22	Best Model Stepwise Forward	Best Adjusted R² In: P value 0.05 Out: P Value 0.10 P value 0.05	0.37 0.23 0.22	0.33 0.22 0.20	60 65 66	55.3 59.3 59.2	78.0 63.0 61.6	WE = 189.6+5.63×ENSO-15.12×NAO-2.03×ERA Tp-0.11×ERA Ap+2.42×ERA Tp GL5P+0.068×ERA Ap M1-2.17×ERA Ap M4+0.055×ERA Ap M5 WE = 184.9-0.96×ERA Tp M1+0.73×ERA Tp WE = 183.3-16.08×NAO-0.77×ERA Tp M1+0.53×ERA Tp M3	8 2 3	ERA Tp ERA Tp M1 ERA Tp M1
	Years, ENSO, NAO, IOD, ERA Tp, ERA Ap, APH Tp and APH Ap	40	**Best Model** Stepwise Forward	Best Adjusted R² In: P value 0.05 Out: P Value 0.10 P value 0.05	**0.51** 0.44 0.39	0.43 0.40 0.36	138 144 143	**48.1** 47.9 50.1	92.5 88.6 77.0	WE = 172.7-23.3×NAO-1.30×ERA Tp M1+1.11×ERA Tp M3+12.41×APH Tp GL5-11.16×APH Tp GL5D+12.76×APH Ap M4-0.039×APH Ap M5+0.24×APH Ap GL5-0.13×APH Ap GL5P-0.18×APH Ap GL5D WE = 183.9-0.75×ERA Tp M1+2.81×ERA Tp M3-3.07×ERA Tp GL5P+3.92×APH Tp M4-1.62×APH Tp M5 WE = 182.5-1.05×ERA Tp M1+0.70×ERA Tp M3+3.67×APH Tp M4-1.57×APH Tp M5	10 5 4	**APH Ap GL5P** APH Tp M4 ERA Tp M1

Three different regression models’ approaches are employed using different number of predictor variables at monthly scale. Difference in number of predictors for the Tarbela and Mangla is due to different number of sub-basins. Best models are showed in bold (with highest R^2^ value). Most influential predictor is also identified based on its maximum contribution to model performance and is displayed in bold in extreme column.

During MLRs model development, all possible predictor variables-based combinations are evaluated to assess their individual role as well as their combined role in WEs predictions. These combinations are (i) COs (ii) ERA Tp and Ap (iii) APH Tp and Ap (iv) ERA + APH Tp and Ap (v) COs+ ERA Tp and Ap (vi) COs+ APH Tp and Ap and (viii) All predictor variables. Our resultant MLR model presented in Table 1 is the best one and showed no higher relationship as compared to models based on any of the above predictor’s combinations.

The above presented WEs prediction models provide real time information to be incorporated into water accounting and sharing plan. Though, the accuracy of the models is limited, nevertheless the developed models hold merit to be considered for further engagement of variables in future research studies. Inclusion of the COs nevertheless in model is based on the fact that the global COs are modulating the weather systems responsible for the precipitation regimes in the upper Indus Basin. The study thus suggests that there are connections between the reservoir’s WE and Tp and Ap in the respective basins. Thus, WEs are predicted somewhat skillfully for better water management.

## Discussion

The presented analysis provides an independent (remotely sensed) information set about WEs of the reservoirs at both monthly and seasonal scales which was previously not available for irrigation water distribution for crops production downstream. We examined the upstream precipitation behavior of both the reservoirs to link WE behavior with it. Interestingly, ERA based Tp and APH based Ap show a significant decreasing trend. This highlights that both Tp and Ap are inherently different as one is modeled and the other one is based on a dense network of meteorological stations.

COs play a role in a hydrological cycle at global, regional and basin scales^37^. The ENSO is a complex climate system which demands improvements in climate related products and services to secure billions of population against vulnerability to natural disasters^38^. Climate system of Pacific Ocean had faced three major El Niño events in the 20th Century i.e., 1997–1998, 1982–1983 and 1925–1926^39^. These COs are responsible for specific weather regimes on land at global and regional scales by influencing the precipitation patterns, temperature regimes, and atmospheric pressures changes. Winter precipitation in western Himalaya is linked to NAO affecting WDs^40^. A large amount of precipitation in western Himalayas is governed by positive NAO phase as compared to lower amount in negative NAO phase based on ERA-40 and 20CR precipitation datasets^41^.

Mangla reservoir WE variability is different from Tarbela as its basin is situated in peak MS belt and less influenced by the WD. However, Tarbela reservoir WE is equally influenced by WD and MS precipitation. The ENSO, NAO and IOD are observed to directly and indirectly impact the precipitation weather system at 6–12 months in advance at global scale as compared to WEs with 1–3 months at local scale^39^. Annual cumulative SSTA based indices classification scheme indicates higher variability for ENSO as compared to NAO and IOD. All three COs are found to be associated with flooding events in Pakistan which identify the interactions among them. Drought is found to be more influenced by the ENSO and IOD signifying the role of summer season.

Sub-basin analyses are carried out to identify hotspot sub**-**basins as well as hotspot seasons in Tarbela and Mangla basins in term of changing Tp and Ap. The ERA Tp trends show more significant decrease in three sub**-**basins (26840 km^2^) of Mangla and five sub**-**basins of Tarbela (18479 km^2^) during Post WD as compared to Pre MS which affected only three sub**-**basins of Gilgit for Tarbela (12743 km^2^). In contrast, all sub**-**basins of Tarbela are showing a decrease in APH Tp during Post WD as compared to one sub**-**basin of Mangla. The Post WD season is identified as the one displaying most changing Tp for Tarbela as well as for Mangla which could be a reason of early water shortage for summer crops. Both ERA and APH based Ap are showing even more decreasing trends than Tp. All sub**-**basins of Tarbela and Kunhar, Neelum and Poonch sub**-**basins of Mangla are showing a decrease in spatial extent of precipitation during Post WD season. The Jhelum sub**-**basin is found as changing hotspot of Mangla basin both for WD as well as for MS Tp. The sub**-**basins of Gilgit, Hunza, Shingo, upper Indus, Zanksar and Shyok are acting as hotspots in term of Ap decrease.

The MLRs are developed for prediction of WEs to provide an early information to water planning and management stakeholders to ensure irrigation water supply downstream around critical growth stage of crops. Prior to this study, water supply is planned based on short to long term weather forecasting only. This study suggests that there is connection between the WEs and upstream Tp and Ap. It is reported that water accounting by Indus river system authority^13^ does not take into account precipitation, evaporation and other information essential for water accounting^25,42^. Briscoe and Qamar indicated that Pakistan water apportionment accord of 1991 needs well calibrated systems for water storage reservoirs monitoring, river flows and transparent along with real time reporting^43,44^. Overall, IRB water balance lacks accuracy and large amount of water remains unaccounted. Remotely sensed data availability has increasing capacity in recent past to provide vital information to assess the poorly monitored IRB^45^. This research study will add on to possible solution related to better IRB water resource management by providing relevant local climate model construction being identified as one of knowledge gap in newly approved National Water Policy 2018 of Pakistan^23^.

## Conclusions

We pointed out that Landsat satellites-based observed WE of reservoirs for the period of 1981–2017 are an indicator of water storage status. These are linked with precipitation amount, and surface area receiving the precipitation in upstream basins, along with climatic oscillation specific sea surface temperature anomalies. These satellites-based WE assessments will ensure the downstream water and food security to millions of people inside Pakistan by integrated incorporation of available forecasts of precipitation, spatial area receiving this precipitation inside basins of these reservoirs, along with ENSO, NAO and IOD based SSTA anomalies, as pointed out in this study. Previously, such linking is only partially explored in the form of selected stream inflow simulations at specific gauge points based on hydrological models.

A range of WE models are developed and presented in this study. Using the multiple linear regression models that best explain the variability in WE, the relevant stakeholders can estimate the WE year-round for water management and for research and development. Precipitation behavior in Tarbela sub-basins T3 (2283.2 km^2^ with 10.7 km^2^ of glaciated area) and T25 (7201.1 km^2^ with 179.7 km^2^ of glaciated area) is dominantly relevant for WE variability in Tarbela reservoir. Similarly, precipitation behavior in sub-basin of GL5P (17327.0 km^2^ with 180.8 km^2^ of glaciated area) is more pertinent to WE variability in Mangla reservoir, situated in region of Pakistan. Given the considerable multifaceted challenges related to long term weather monitoring in high mountain areas, our obtained results in this study provide information for policy makers/managers for sub-basins where more careful weather monitoring is suggested out of all sub-basins for each reservoir, separately.

## Methods

The IRB is spread across four different countries of Pakistan, India, Afghanistan and China, of which major part lies in Pakistan followed by India, and the other two countries^11^. We selected the study area covering two major water reservoirs in Pakistan constructed on rivers of Indus and Jhelum, which are a major source of water (Fig. 1). Every reservoir receives water from a particular basin area and boundary delineation is a primary requisite for research related to hydrology, water resource management, climate change impacts, agricultural water productivity, and environmental and watershed management^46–52^.

We used an integrated approach for this study (Extended Fig. 4). Satellite data based digital elevation models (DEMs) has been widely used for basin boundary delineation using manual to automatic procedures^53,54^. We used ALOS PRISM 30 m resolution global DEM for basin delineation based on semi-automatic basin and sub-basins extraction approach called soil and water analysis tool (SWAT)^55^. This SWAT tool uses flow direction analysis based on D8 flow routing algorithm^56,57^. The extracted basin boundary after refinements represents around 170,600 km^2^ and lie in the upper IRB under study. Pakistan’ PUIRB is spread across 96,700 km^2^ and out of it 19,100 km^2^ is glaciated with around 8000 glaciers^58,59^. Rest of the PUIRB basin lies in disputed area of Kashmir with an area of 73,900 km^2^ that includes glaciated area of 6,600 km^2^ with 4900 glaciers. The PUIRB basins consist of a number of sub**-**basins and are documented for both reservoirs and various rivers basins (Extended Table 1). The Tarbela-Indus basin consists of 26 sub**-**basins linked with 9 sub**-**basins of Gilgit, Hunza, lower part of upper Indus, Shigar, Astore, Shyok, Shingo, Zanskar and upper Indus (138,505 km^2^). Tarbela specific basin is fed by 14 different rivers of Gilgit, Khunjrab, Shimshal, Indus, Hunza, Shigar, Astore, Shyok, Nubra, Hushey, Shingo, Dras, Suru and Zanskar. The Mangla**-**Jhelum consists of five sub**-**basins linked to five rivers of Neelum, Jhelum, Kunhar, Kanshi and Poonch rivers (32,095 km^2^).

We used annual, seasonal (Meteorological or MET and Cropping or AGR) and monthly time scales for the current study. Summary of the specific seasons is presented in Extended Table 5. The MET seasons in Pakistan are characterized by two major weather systems which are WD linked to winter precipitation and MS system linked to summer precipitation. Irrigation water distribution through reservoirs is adjusted in view of the two cropping seasons of Kharif (summer) and Rabi (spring). Cotton, rice, sugarcane and maize are major summer crops, while wheat is most dominating spring crop in Pakistan^60,61^.

### Landsat satellite data

The Landsat satellite 3, 5, 7 and 8 data covering period of 1981–2017 are used to extract the WEs of both reservoirs (Extended Table 6). Landsat grid number 150/36 covers Tarbela reservoir while grid number149/37 covers Mangla reservoir. A total of 404 cloud free Landsat satellite images are downloaded and processed using an open sources QGIS tool. The normalized difference water index (NDWI) is computed using modified NDWI to detect the WE of reservoirs^62^. All NDWI images are subsetted using reduced area of interest (AOI) to optimize the size of the images for efficient processing based on developed ArcGIS models. The NDWI threshold of 0.00–0.20 is used to extract the WE more effectively in the form of binary raster (1 = water and 0 = no water) as recently proposed^63,64^. The NDWI binary images are further vectorized to get the WE. All peak storage WEs of August to October months are used to establish both reservoirs’ maximum boundary. All AOI specific multi-temporal WEs contain some water features outside the reservoir site and are removed using vector clipping approach developed in ArcGIS model environment. The Landsat 7 satellites scan line corrector (SLC) error is corrected in vector layer by using polygons aggregation tool in ArcGIS (see Section on Codes and ArcGIS model) instead of using gap filling approach in raster domain. We measured the approximate size of missing data line width size and used a distance parameter of 320–360 m for error correction. The Mangla WE is revised by subtracting approx. 58.3 km^2^ from computed WE after 2009 till 2017 due to Mangla raising project (2004–2009).

### Gridded precipitation data

Gridded precipitation datasets may be used as proxy to *in situ* data for ungauged locations with varying cons and pros^64^. The APH data with 0.25° × 0.25° spatial resolution is used for the period of 1981–2007^65,66^. The APH precipitation data is based on dense rain gauge network of 5000–12000 stations and is primarily generated through distance weighting spatial interpolation method. We downloaded the APHRO MA V1101 data spread across 60° E–150° E, 15° S–55° N. The APH precipitation data set is selected because of its fine spatial resolution and performance relative to several other existing gridded precipitation data sets available for Pakistan^67–69^, as well as in similar climatic conditions^70,71^. The daily ERA precipitation data with 0.25° × 0.25° spatial resolution are also employed^72^. The ERA Web API and python procedure is used for downloading the daily precipitation (parameter = 228.128) in annual netcdf file format. Both gridded precipitation data are extracted based on reservoir’s specific basin extents using ArcGIS automatic data extraction tool in the database format (dbf). These daily precipitation dbf files are converted to annual files using Visual Basic macro developed using Microsoft excel.

The APH and ERA precipitation variables of Tp and Ap at monthly, seasonal and annual time scales are computed using Eqs. (1 and 2), respectively.

1 $$Tp={p}_{d1}+{p}_{d2}+{p}_{d3}+\ldots +{p}_{dn}$$

where *p*_*d*1_ is the observed precipitation amount on day 1 and so on. The Ap is based on summing up the grid box areas of 0.25° × 0.25° or 625 km^2^, where the observed precipitation exceeds the threshold of 0.1 mm day^−1^. An objective definition of threshold is difficult and depends on spatial resolution of the data^73^:

2 $$Ap=\sum _{i}\,{a}_{i}H({p}_{i}-{x}_{0})$$

where *a*_*i*_ is the area of grid box, *p*_*i*_ is daily precipitation and *x*_0_ is the threshold criterion. The ${\rm{H}}(\,\cdot \,)$ is the Heaviside function in Eq. (2).

### CO indices

We assessed the statistical relationship between the reservoir’ WEs variability and COs indices of ENSO, NAO and IOD based on SSTA^37,74,75^. Three month running average approach is used to compute the SSTA anomaly. Selection of specific indices are based on sensitivity of winter and summer precipitation in PUIRB of Pakistan and have already been used in numerous earlier studies^76,77^. The WEs of reservoirs are found to be related with SSTA based indices as evidenced via correlation analysis. The annual CO classification scheme has been proposed using cumulative SSTA by developing 11 classes based on percentiles taking into account negative/positive phases of the oscillations that prevailed throughout 1981–2017, following ref. ^75^ (Extended Fig. 3, Extended Table 7).

### Sensitivity and statistical analysis

Annual trends of Tp and Ap are computed for each reservoir’ basin using linearly regressed fits during the period of 1981–2017^77^. These trends are also linked with identified ENSO positive and negative phase years^78^. The time series data of hydrological nature can be statistically analyzed using parametric or non-parametric trend tests to identify the underlying trends^79^. The trends in Tp, Ap, monthly and seasonal indices of ENSO, NAO and IOD are computed using nonparametric MK test and Sen’s slope at *p* ≤ 0.05 and also addressed the issue of serial correlations and biasness for Mangla, Tarbela basins and overall upper IRB^80–82^. Further, sensitivity analysis for linear relationship of reservoirs’ WE with Tp, Ap, three CO indices as well as between precipitation and COs is performed by utilizing Pearson correlation coefficient (CC) based on Eq. (3)^83,84^:

3 $$C{C}_{WE,P}=\frac{{\sum }_{i=1}^{n}\,(W{E}_{i}-\overline{WE})({P}_{i}-\bar{P})}{\sqrt{{\sum }_{i=1}^{n}\,{(W{E}_{i}-\overline{WE})}^{2}}\times \sqrt{{\sum }_{i=1}^{n}\,{({P}_{i}-\bar{P})}^{2}}}$$

where *i* represents the observed value, and overbar is the mean observation value. The P represents precipitation. The correlations among WE and CO are computed by replacing P with CO in Eq. (3).

### Decadal change analysis (1981–2017)

Long term decadal change analysis (DC) is performed for four consecutive period of 1981–1990, 1991–2000, 2001–2010 and 2011–2017 with respect to 37 years median value (Extended Fig. 1):

4 $$D{C}_{ERATp}=\frac{ERAT{p}_{i}-ERAT{p}_{1981-2017}}{ERAT{p}_{(1981-2017)}}$$

where *i* represents the given minimum value observed for a decade and denominator is a median value for 37 years. The DC for ERA Ap, APH Tp, APH Ap and WE are computed by replacing ERA Tp with these variables in Eq. (4) and so on. Two sample t-test is applied to evaluate the decadal change in mean of WE with respect to normal WE of both reservoirs^84^.

### Combined impact of precipitation and COs on WEs

**Figure 7:**
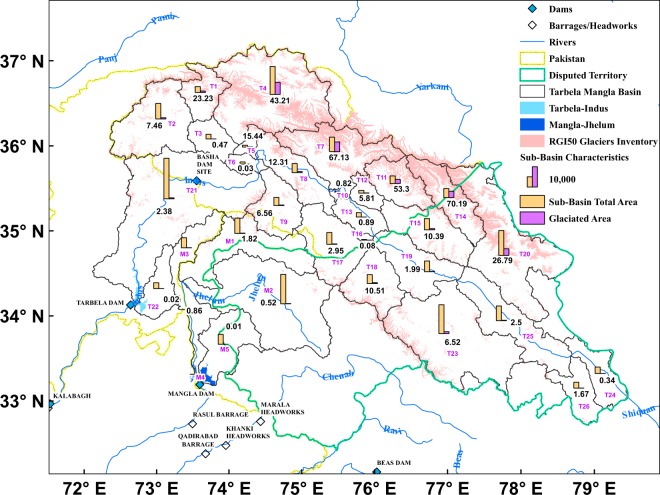
Sub-basin characteristics based on distribution and size of glaciated areas using Rudolph glacier inventory. The glaciated area percent contribution at sub**-**basin scale is used to generate new predictor variables for multiple linear regression models for WE prediction. We have addressed the following question: whether precipitation of glaciated sub**-**basins play some role in WE variability or not? Detailed information is provided in Extended Table 1. Glacier inventory data was downloaded from http://www.glims.org/RGI/randolph50.html (RGI Consortium (2015). Randolph Glacier Inventory -A Dataset of Global Glacier Outlines: Version 5.0: Technical Report, Global Land Ice Measurements from Space, Colorado, USA. Digital Media. https://doi.org/10.7265/N5-RGI-50).

We also examined the co-variability of WE of reservoirs with Tp, Ap and three COs along with yearly trends based on MLRs (Table 1, Extended Table 8). Before developing model, we incorporated the new precipitation related variables based on glaciated area at sub-basin and its location in Pakistan or disputed areas (Fig. 7). Three variable classes are established which are GL5 (precipitation from glaciated area less than 5% of sub-basin total area), GB520 (precipitation from glaciated area between 5 – 20% of sub-basin total area) and GG20 (precipitation from glaciated area greater than 20% of sub-basin total area). These three classes are further categorized based on precipitation received as overall basins (GL5, GB520 and GG20), within Pakistan (GL5P, GB520P and GG20P) and the disputed areas that lie in basins (GL5D, GB520D and GG20D). Initially, developed regression models are subject to two statistical procedures of PCA and multi-collinearity testing. The PCA is employed to reduce the dimensionality of a large number of interrelated variables and to retain the dominant variations present in the data^84,85^. Multi-collinearity among independent variables is examined based on correlation coefficient matrix analysis^84^. Three approaches are employed to find the best model for the prediction of WE variability. These are best (parameters are minimum variable, maximum variable and adjusted R^2^), stepwise (independent variable in and out based on probability criteria of in 0.05 and out 0.10) and forward (same as stepwise except for the variable is added only). The validation is carried out based on random 15 observations from the matrix. Best regression model is selected based on highest R^2^ value and lowest root mean square error (RMSE)^83^:

5 $$RMSE\,(k{m}^{2})=\frac{1}{n}\sqrt{\mathop{\sum }\limits_{i=1}^{n}\,(W{E}_{pre}-W{E}_{obs})}$$

where WE_pre_ (WE_obs_) stands for predicted WEs (observed WEs).

### Codes and ArcGIS model

Landsat satellite data calibrations and NDWI is computed by using RSGIS toolbox^86^ in QGIS ver 2.18.17 software (https://qgis.org/en/site/forusers/download.html and https://github.com/PrathamGitHub/NITK_RS-GIS_17/blob/master/RSGIS_M.py); Landsat 7 SLC error is corrected using polygon aggregation (https://www.ian-ko.com/ET_GeoWizards/UserGuide/aggregatePolygons.htm); ERA interim data is downloaded automatically by using ERA Web API (https://confluence.ecmwf.int/display/WEBAPI/Web-API+Downloads). We also used SDM toolbox for the conversion of netcdf into tif format (http://sdmtoolbox.org/downloads) and developed a number of GIS models for basic processing like automatic clipping, data extraction and others. Similarly, hundreds of dbf extracted files are compiled into single Excel file based on developed VB macro. All GIS models and codes are available on request.

## Supplementary information

Supplementary information 

## Data Availability

We have used the following published datasets: Landsat satellite data (https://earthexplorer.usgs.gov/); ALOS 30 m GDEM (http://www.eorc.jaxa.jp/ALOS/en/aw3d30/); APHRODITE APHRO_MA_025_V1101 (http://www.chikyu.ac.jp/precip/english/products.html); ERA Interim Reanalysis data (http://apps.ecmwf.int/datasets/data/interim-full-daily/levtype=sfc/); Randolph RGI50 (version 5, http://www.glims.org/RGI/); ENSO monthly SSTA data (https://www.esrl.noaa.gov/psd/gcos_wgsp/Timeseries/Data/nino34.long. anom.data); NAO monthly SSTA data (https://www.esrl.noaa.gov/psd/gcos_wgsp/Timeseries/Data/nao.long.data); IOD monthly SSTA data (http://www.jamstec.go.jp/frcgc/research/d1/iod/e/iod/dipole_mode_index.html).

## References

[1] Immerzeel WW, Bierkens MFP (2012). Asia’s water balance. Nature Geoscience.

[2] Immerzeel WW (2015). Reconciling high-altitude precipitation in the upper Indus basin with glacier mass balances and runoff. Hydrology and Earth System Sciences.

[3] Pritchard HD (2019). Asia’s shrinking glaciers protect large populations from drought stress. Nature.

[4] Bolch T (2017). Asian glaciers are a reliable water source. Nature.

[5] Immerzeel WW (2010). Climate change will affect the Asian water towers. Science.

[6] Siebert S (2005). Development and validation of the global map of irrigation areas. Hydrology and Earth System Sciences.

[7] Biemans H (2011). Impacts of reservoirs on river discharge and irrigation water supply during 20th century. Water Resources Research.

[8] Johannis, M., Flint, L. E., Dettinger, M. D., Flint, A. L. & Ochoa R. The role of snowpack, rainfall, and reservoirs in buffering California against drought effects. *Fact Sheet 2016–3062*, USGS, USA (2016).

[9] Ho M (2017). The future role of dams in the United States of America. Water Resources Research.

[10] Lutz, A. *et al*. Development of baseline climate dataset and trend analysis in the Mekong river basin. *Future Water Report version 14*, Netherlands, 127 pages (2014).

[11] Laghari AN, Vanham D, Rauch W (2012). The Indus basin in the framework of current and future water resources management. Hydrology and Earth System Sciences.

[12] Keller, A. Sakthivadivel, R. & Seckler, D. Water Scarcity and role of storage in development. Research Report 39, IWMI, Sri Lanka, 20 pages (2000).

[13] Indus River System Authority (IRSA). Irrigation and rivers network of Pakistan, http://pakirsa.gov.pk/ (Accessed on 01 September, 2019).

[14] Ahmad B (2011). Water Management: A solution to water scarcity in Pakistan. Journal Independent Studies and Research.

[15] Usman M (2016). Contribution of agriculture sector in the GDP growth rate of Pakistan. Journal of Global Economics.

[16] Ahmad, S. Water Insecurity: A threat for Pakistan and India. Issue Brief, South Asia Centre, Atlantic Council, Washington (2012).

[17] Grigg N (2018). The water-food-energy nexus in Pakistan: a biophysical and socio-economic challenge. Proceedings of the International Association of Hydrological Sciences.

[18] The World Bank. Better management of Indus basin waters; Strategic issues and challenges. The World Bank, 8 pages, www.worldbank.org.pk (2006).

[19] International Monetary Fund (IMF). Issues in managing water challenges and policy instruments: Regional perspectives and case studies, https://www.imf.org/external/pubs/ (2005).

[20] Young, W. *et al*. Pakistan: getting more from water. Water Security Diagnostics. Washington, D.C, http://documents.worldbank.org (2019).

[21] Karki MB, Shrestha AB, Winiger M (2011). Enhancing knowledge management and adaptation capacity for integrated management of water resources in the Indus river basin. Mountain Research and Development.

[22] Government of Pakistan. 6th Population and Housing Census 2017. Pakistan Bureau of Statistics, http://www.pbs.gov.pk (Accessed on 01 September, 2019).

[23] Government of Pakistan. Pakistan National Water Policy 2018. *Ministry of water resources*, (2018), https://www.ffc.gov.pk/ (Accessed on 01 September, 2019).

[24] Kahlown MA, Majeed A (2002). Water resources situation in Pakistan: challenges and future strategies. Science Vision.

[25] Yu, W. *et al*. The Indus basin of Pakistan: the impacts of climate risks on water and agriculture. *World Bank Report*, USA (2013).

[26] Government of Pakistan. Daily hydrological data. Hydrology and water management organization, Water and Power Development Authority, https://www.wapda.gov.pk (Accessed on 01 September, 2019) (2019).

[27] Ahmad, F. Water scarcity, Dawn, https://www.dawn.com/news/1410709 (2018).

[28] Liu H, Yin J, Feng L (2018). The dynamics changes in the Storage of the Danjiangkou reservoir and the influence of the south-north water transfer project. Scientific Reports.

[29] Briscoe J (2009). Water security: why it matters and what to do about it. Innovations: Technology, Governance, Globalization.

[30] Baldassarre GD (2018). Water shortage worsened by reservoir effects. Nature Sustainability.

[31] Arjasakusuma S (2018). ENSO and rainfall sensitive vegetation regions in Indonesia as identified from multi-sensor remote sensing data. ISPRS International Journal of Geo-Information.

[32] Lehner B (2011). High-resolution mapping of the world’s reservoirs and dams for sustainable river-flow management. Frontiers in Ecology and the Environment.

[33] Yang Y (2014). Water governance and adaptation to climate change in the Indus River Basin. Journal of Hydrology.

[34] Cai X, Lian F, Xuejiao H, Xiaoling C (2016). Remote sensing of the water storage dynamics of large lakes and reservoirs in the Yangtze River Basin from 2000 to 2014. Scientific Reports.

[35] Speed, R. *et al*. Basin water allocation planning: Principles, procedures and approaches for basin allocation planning, https://www.adb.org/ (Accessed on 01 September, 2019) (2013).

[36] Anwar AA, Bhatti MT (2018). Pakistan’s water apportionment Accord of 1991: 25 years and beyond. Journal of Water Resources Planning and Management.

[37] Timmermann A (2018). El Niño-Southern oscillation complexity. Nature.

[38] Takahashi K, Martínez AG (2019). The very strong coastal El Niño in 1925 in the far-eastern Pacific. Climate Dynamics.

[39] Quinn WH, Neal VT (1987). El Niño occurrences over the past four and a half centuries. Journal of Geophysical Research.

[40] Saeed SF, Giorgi F, Pal JS, Keay K (2010). Regional climate model simulation of winter climate over Central–Southwest Asia, with emphasis on NAO and ENSO effects. International Journal of Climatology.

[41] Filippi L (2014). Multidecadal variations in the relationship between the NAO and winter precipitation in the Hindu Kush-Karakoram. Journal of climate.

[42] Yang Y (2013). An introduction to the IBMR, a hydro-economic model for climate change impact assessment in Pakistan’s Indus River basin. Water International.

[43] Briscoe, J. & Qamar, U. Pakistan’s water economy: Running dry, Oxford University Press, Oxford, U.K (2005).

[44] Government of Pakistan. Apportionment of waters of Indus River System between the provinces of Pakistan, https://waterinfo.net.pk/ (Accessed on 01 September, 2019) (1991).

[45] Hasson, S. & Böhner, J. Hydrological cycle over Indus Basin at Monsoon Margins: Present and Future. In: *Indus River Basin*, Elsevier, pp. 183–201 (2019).

[46] Khan A (2011). How large is the upper Indus basin? The pitfalls of auto-delineation using DEMs. Journal Hydrology.

[47] Chien H, Yeh PJ-F, Knouft JH (2013). Modeling the potential impacts of climate change on streamflow in agricultural watersheds of the midwestern United States. Journal of Hydrology.

[48] Shrestha RR, Dibike YB, Prowse TD (2012). Modelling of climate-induced hydrological changes in the lake Winnipeg watershed. Journal of Great Lakes Research.

[49] Park J-H (2010). Potential effects of climate change and variability on watershed biogeochemical processes and water quality in northeast Asia. Environment International.

[50] Faramarzi M (2013). Modeling impacts of climate change on fresh water availability in Africa. Journal Hydrology.

[51] Stakhiv E, Stewart B (2010). Needs for climate information in support of decision making in the water sector. Procedia Environmental Science.

[52] Ficklin DL (2009). Climate change sensitivity assessment of a highly agricultural watershed using SWAT. Journal Hydrology.

[53] Luo Y (2013). Assessment of climate change impacts on hydrology and water quality with a watershed modeling approach. Science of the Total Environment.

[54] Jenson SK, Domingue JO (1988). Extracting topographic structure from digital elevation data for geographic information system analysis. Photogrammetric Engineering and Remote Sensing.

[55] Tarboton DG, Bras RL, Rodriguez-Iturbe I (1991). On the extraction of the channel networks from digital elevation data. Hydrological Processes.

[56] Tadono, T. *et al*. Generation of the 30 m mesh global digital surface model by ALOS PRISM. *The international archives of the photogrammetry*, *remote sensing and spatial information sciences*, Volume XLI-B4, 2016 XXIII ISPRS Congress, 12–19 July 2016, Prague, Czech Republic, 158–162 (2016).

[57] Mark DM (1984). Automated detection of drainage networks from digital elevation models. Cartographica.

[58] O’Callaghan JF, Mark DM (1984). The extraction of drainage networks from digital elevation data. Computer Vision, Graphics, Image Processing.

[59] Pfeffer WD (2014). The Randolph glacier Inventory: a globally complete inventory of glaciers. Journal of Glaciology.

[60] Bussay, A. & Akhtar, I. H. Wheat yield/production forecasting and estimation technology. SUPARCO, Islamabad, Pakistan (2008).

[61] Bussay, A. & Akhtar, I. H. Crop yield/production forecasting and estimation technology for kharif crops (cotton, rice & sugarcane). SUPARCO, Islamabad, Pakistan (2009).

[62] Xu H (2006). Modification of normalized difference water index (NDWI) to enhance open water features in remotely sensed imagery. International Journal of Remote Sensing.

[63] Huang C (2018). Detecting, extracting, and monitoring surface water from space using optical sensors: A review. Reviews of Geophysics.

[64] Dahri ZK (2016). An appraisal of precipitation in the high-altitude catchments of the Indus basin. Science of the Total Environment.

[65] Yatagai A (2009). A 44-year daily precipitation dataset for Asia based on a dense network of rain gauges. Scientific Online Letters on the Atmosphere.

[66] Yatagai A (2012). APHRODITE, constructing a long term daily gridded precipitation dataset for Asia based on a dense network of rain gauges. Bulletin of the American Meteorological Society.

[67] Rana S, McGregor J, Renwick J (2015). Precipitation seasonality over the Indian subcontinent: an evaluation of gauge, reanalyses, and satellite retrievals. Journal of Hydrometeorology.

[68] Nabeel A, Athar H (2018). Classification of precipitation regimes in Pakistan using wet and dry spells. International Journal of Climatology.

[69] Ceglar A, Toreti A (2017). Precipitation over monsoon Asia: a comparison of reanalyses and observation. Journal of Climate.

[70] El Kenawy AM, McCabe MF (2016). A multi-decadal assessment of the performance of gauge and model-based rainfall products over Saudi Arabia: climatology, anomalies and trends. International Journal of Climatology.

[71] Tan ML, Gassman PW, Cracknell AP (2017). Assessment of three long term gridded climate products for hydro-climatic simulations in tropical river basins. Water.

[72] Dee DP (2011). The ERA-Interim reanalysis: configuration and performance of the data assimilation system. Quarterly Journal of the Royal Meteorological Society.

[73] Benestad RE (2018). Implications of a decrease in the precipitation area for the past and the future. Environmental Research Letters.

[74] Saji NH (1999). A dipole mode in the tropical Indian ocean. Nature.

[75] Hurrell JW (1995). Decadal trends in the north Atlantic oscillation: regional temperatures and precipitation. Science.

[76] Yuan C, Yamagata T (2015). Impacts of IOD, ENSO and ENSO Modoki on the Australian winter wheat yields in recent decades. Scientific Reports.

[77] Ward PJ (2014). Strong influence of El Nino southern oscillation on flood risk around the world. Proceedings of the National Academy of Sciences of the United States of America.

[78] Matias H (2018). Two thirds of global cropland area impacted by climate oscillations. Nature Communications.

[79] Farhan SB (2015). Hydrological regimes under the conjunction of westerly and monsoon climates: a case investigation in the Astore basin, northwestern Himalaya. Climate Dynamics.

[80] Hamed KH, Rao AR (1998). A modified Mann-Kendall trend test for autocorrelated data. Journal of Hydrology.

[81] Hamed KH (2008). Trend detection in hydrologic data: the Mann–Kendall trend test under the scaling hypothesis. Journal of Hydrology.

[82] Hamed KH (2009). Enhancing the effectiveness of prewhitening in trend analysis of hydrologic data. Journal of Hydrology.

[83] Iqbal MF, Athar H (2018). Validation of satellite based precipitation over diverse topography of Pakistan. Atmospheric Research.

[84] Wilks, D. S. Statistical methods in the atmospheric sciences, 3rd Edition. Academic Press, USA (2011).

[85] Jolliffe, I. T. Principal component analysis, 2nd Edition. Springer series in Statistics, USA (2002).

[86] Barane, P. K. & Dwarakish, G. S. Development of a tool for land surface temperature retrieval from Landsat data products. Proceedings of International Conference on Global Civil Engineering Challenges in Sustainable Development and Climate Change, 17–18 March, 2017, India, 195–199 (2017).

