# Supplementary material for: Contribution of changing precipitation and climatic oscillations in explaining variability of water extents of large reservoirs in Pakistan
Source: Sci Rep. 2019 Dec 13;9:19022. doi: 10.1038/s41598-019-54872-x (PMC6910943; doi:10.1038/s41598-019-54872-x)
Supplement: Supplementary file 1 — Supplementary information [file 41598_2019_54872_MOESM1_ESM.docx]

**Contribution of changing precipitation and climatic oscillations in explaining variability of water extents of large reservoirs in Pakistan**

Ibrar ul Hassan Akhtar^1,3^ and H. Athar^1,2,*^

^1^Department of Meteorology, COMSATS University Islamabad, Pakistan

^2^Centre for Climate Research and Development, COMSATS University Islamabad, Pakistan

^3^Space Applications & Research Complex, Pakistan Space and Upper Atmosphere Research Commission, Islamabad, Pakistan

**Supplementary Information**

­­­­_________

Correspondence: Dr. H. Athar, Department of Meteorology, COMSATS University Islamabad, Park Road, Tarlai Kalan, Islamabad 45550, Pakistan. *e-mail: athar.hussain@comsats.edu.pk

**List of Extended Tables**

**Extended Table 1. Same as Fig. 7 except for more details on sub-basins sizes and associated rivers**. Lower part of upper Indus and Shyok basins are largest sub-basins in Tarbela-Indus watershed and spread across 12 sub-basins. Jhelum and Neelum sub-basins are the largest in Mangla-Jhelum watershed.

**Extended Table 2. Decadal changes observed for WEs and ERA and APH based Tp and Ap at seasonal scale.** The t-test statistics show variability in WEs, ERA and APH Tp, AP for both reservoirs. This may be due to synoptic and temporal changes of precipitations modulated by COs in mountainous regions of Himalaya and Karakoram and increased number of reservoirs on tributaries of Indus and Jhelum outside Pakistan. Bold values indicate significance at *p*<0.05.

**Extended Table 3. The MK and Sen’s temporal trend analysis along with Sen’s slope estimates for three month running average SSTA during 1981–2017**. Filled cells with bold values represent the significant trends at 95% confidence level. The revised values reflect the influence of serial correlation.

**Extended Table 4. Same as Extended Table 2 except for MET and AGR seasons (see text for more details)**.

**Extended Table 5. Two different classification schemes are used to evaluate the WEs.** Meteorological seasons take into account the two major weather systems responsible for precipitation in the study area. Cropping seasons are to link the WEs and weather systems with low riparian downstream of Tarbela-Indus and Mangla-Jhelum.

**Extended Table 6. Landsat 3, 5, 7 and 8 satellite data are used for the extractions of the WEs for Tarbela and Mangla reservoirs**. Temporal frequency cover for Landsat 3 is during 1981–83; for Landsat 5 during 1984–2001 & 2008–2011; for Landsat 7 during 2001–2007 & 2012–2013 and for Landsat 8 during 2013–2017.

**Extended Table 7. Same as Extended Fig. 3 except for more detailed information on COs related statistics and floods/drought years recorded in Pakistan.**

**Extended Table 8. List of variables used in development of the multiple linear regression models for Tarbela and Mangla reservoirs.** See text for more details.

**List of Extended Figures**

**Extended Fig. 1. Same as Extended Table 2 except for percent decadal changes.** Analysis shows an alarming decrease in WEs for both reservoirs.

**Extended Fig. 2. Relationship of Tp, Ap with ENSO, NAO and IOD for Tarbela, Mangla and overall basin based on monthly correlation coefficients.** The ENSO shows more strong and significant correlations with Tp and Ap as compared to NAO and IOD.

**Extended Fig. 3. Annual COs classification scheme based on percentiles applied to cumulative SSTA for ENSO, NAO and IOD**. This scheme has identified extreme dry and wet years and is used to link flood and drought events across south Asia including Pakistan. Flood events occurrence is increasing significantly since 1981. Extreme wet/dry conditions have been observed for year 1999/2005 (ENSO), 2010/1989 (NAO) and 1992/1997 (IOD).

**Extended Fig. 4. An integrated approach is developed to assess the co-variability between satellites data-based WE with basin wide precipitation and COs.** First step is the extraction of the reservoir’s specific watershed basin from ALOS GDEM and refinements. Two identified Landsat grids covering the reservoirs are downloaded with no cloud cover present over the water reservoir. The NDWI based WE is linked with ERA and APH Tp and Ap along with ENSO, NAO and IOD based SSTA to explain the fact behind the current decreasing trend in water storages. The statistical techniques of linear trends, correlation coefficients, MK trends, percent changes and multiple regression models are employed to quantify the relationships.

**Extended Table 1. Same as Fig. 7 except for more details on sub-basins sizes and associated rivers**. Lower part of upper Indus and Shyok basins are largest sub-basins in Tarbela-Indus watershed and spread across 12 sub-basins. Jhelum and Neelum sub-basins are the largest in Mangla-Jhelum watershed.

| Basin | Sub-basin codes |  | Area (km^2^) | Sub-basin regions | Associated rivers | |
| --- | --- | --- | --- | --- | --- | --- |
| Tarbela-Indus | T1, T2, T3 |  | 12743.1 | Gilgit | | Gilgit |
|  | T4 |  | 13619.8 | Hunza | | Khunjrab, Shimshal and Hunza |
|  | T5, T6, T8, T10, T13, T21, T22 |  | 31664.2 | Lower part of Upper Indus | | Indus |
|  | T7 |  | 7016.8 | Shigar | | Shigar |
|  | T9 |  | 3990.0 | Astore | | Astore |
|  | T11, T12, T14, T15, T20 |  | 26905.9 | Shyok | | Shyok, Nubra and Hushey |
|  | T17, T18 |  | 10174.9 | Shingo | | Shingo, Dras and Suru |
|  | T23 |  | 13911.1 | Zanskar | | Zanskar |
|  | T16, T19, T24, T25, T26 |  | 18479.5 | Upper Indus | | Indus |
|  |  |  |  |  | |  |
| Mangla-Jhelum | M1 |  | 7421.8 | Neelum | | Neelum |
|  | M2 |  | 14397.0 | Jhelum | | Jhelum |
|  | M3 |  | 4991.2 | Kunhar | | Kunhar |
|  | M4 |  | 1285.0 | Kanshi | | Kanshi |
|  | M5 |  | 4823.1 | Poonch | | Poonch |

**Extended Table 2. Decadal changes observed for WEs and ERA and APH based Tp and Ap at seasonal scale.** The t-test statistics show variability in WEs, ERA and APH Tp, AP for both reservoirs. This may be due to synoptic and temporal changes of precipitations modulated by COs in mountainous regions of Himalaya and Karakoram and increased number of reservoirs on tributaries of Indus and Jhelum outside Pakistan. Bold values indicate significance at *p*<0.05.

| Season | Variables | | Decade | | Normal Period | |  | Tarbela | | | |  | | Mangla | | | | | | | | |  |
| --- | --- | --- | --- | --- | --- | --- | --- | --- | --- | --- | --- | --- | --- | --- | --- | --- | --- | --- | --- | --- | --- | --- | --- |
|  |  |  |  |  |  |  |  | Mean Change | t value | | *p value* | |  | | Mean Change | | | t value | | *p value* | |  |  |
| MET | | WE (km^2^) | | 1981-1990 | | 1981-2017 |  | -15.693 | -1.29 | 0.253 | |  | | -26.511 | | -1.713 | | | 0.147 | | | |  |
|  |  |  |  | 1991-2000 | |  |  | **-53.354** | -7.509 | 0.001 | |  | | -69.118 | | -2.106 | | | 0.089 | | | |  |
|  |  |  |  | 2001-2010 | |  |  | **-44.593** | -5.751 | 0.002 | |  | | **-63.125** | | -6.329 | | | 0.001 | | | |  |
|  |  |  |  | 2011-2017 | |  |  | **-23.918** | -3.569 | 0.016 | |  | | **-52.312** | | -4.179 | | | 0.009 | | | |  |
|  |  | ERA Tp (mm) | | 1981-1990 | | 1981-2017 |  | 8.186 | 1.509 | 0.192 | |  | | 8.244 | | 0.523 | | | 0.624 | | | |  |
|  |  |  |  | 1991-2000 | |  |  | 10.64 | 1.77 | 0.137 | |  | | 25.464 | | 1.611 | | | 0.168 | | | |  |
|  |  |  |  | 2001-2010 | |  |  | -15.973 | -2.025 | 0.079 | |  | | -22.774 | | -1.454 | | | 0.214 | | | |  |
|  |  |  |  | 2011-2017 | |  |  | -8.007 | -2.429 | 0.059 | |  | | -15.62 | | -1.533 | | | 0.186 | | | |  |
|  |  | ERA Ap (km^2^) | | 1981-1990 | | 1981-2017 |  | 57.329 | 0.978 | 0.373 | |  | | 23.055 | | 0.264 | | | 0.802 | | | |  |
|  |  |  |  | 1991-2000 | |  |  | 100.13 | 0.8 | 0.419 | |  | | -2.627 | | -0.03 | | | 0.977 | | | |  |
|  |  |  |  | 2001-2010 | |  |  | **-223.821** | -3.524 | 0.017 | |  | | -76.888 | | -1.025 | | | 0.352 | | | |  |
|  |  |  |  | 2011-2017 | |  |  | 15.428 | 0.112 | 0.915 | |  | | 80.658 | | 0.959 | | | 0.381 | | | |  |
|  |  | APH Tp (mm) | | 1981-1990 | | 1981-2007 |  | -0.12 | -0.037 | 0.972 | |  | | -1.248 | | -0.537 | | | 0.614 | | | |  |
|  |  |  |  | 1991-2000 | |  |  | 3.506 | 1.048 | 0.342 | |  | | 3.301 | | 1.274 | | | 0.259 | | | |  |
|  |  |  |  | 2001-2007 | |  |  | -6.763 | -2.01 | 0.101 | |  | | -2.932 | | -1.778 | | | 0.136 | | | |  |
|  |  | APH Ap (km^2^) | | 1981-1990 | | 1981-2007 |  | **228.947** | 3.209 | 0.024 | |  | | **276.038** | | 3.504 | | | 0.017 | | | |  |
|  |  |  |  | 1991-2000 | |  |  | -48.501 | -0.424 | 0.689 | |  | | -48.219 | | -0.506 | | | 0.634 | | | |  |
|  |  |  |  | 2001-2007 | |  |  | **-277.551** | -3.157 | 0.025 | |  | | **-325.457** | | -3.154 | | | 0.025 | | | |  |
| AGR | | WE (km^2^) | | 1981-1990 | | 1981-2017 |  | -23.983 | -2.039 | 0.111 | |  | | -21.906 | | -1.614 | | | 0.182 | | | |  |
|  |  |  |  | 1991-2000 | |  |  | **-45.664** | -5.229 | 0.003 | |  | | -71.949 | | -2.325 | | | 0.068 | | | |  |
|  |  |  |  | 2001-2010 | |  |  | **-43.400** | -5.300 | 0.003 | |  | | **-66.721** | | -5.424 | | | 0.003 | | | |  |
|  |  |  |  | 2011-2017 | |  |  | **-25.295** | -3.033 | 0.039 | |  | | **-46.518** | | -3.491 | | | 0.017 | | | |  |
|  |  | ERA Tp (mm) | | 1981-1990 | | 1981-2017 |  | 8.186 | 1.472 | 0.201 | |  | | 8.244 | | 0.517 | | | 0.627 | | | |  |
|  |  |  |  | 1991-2000 | |  |  | 10.640 | 1.680 | 0.154 | |  | | 25.464 | | 1.529 | | | 0.187 | | | |  |
|  |  |  |  | 2001-2010 | |  |  | -15.973 | -2.362 | 0.065 | |  | | -22.774 | | -1.428 | | | 0.213 | | | |  |
|  |  |  |  | 2011-2017 | |  |  | -8.007 | -1.451 | 0.206 | |  | | -15.620 | | -0.807 | | | 0.456 | | | |  |
|  |  | ERA Ap (km^2^) | | 1981-1990 | | 1981-2017 |  | 38.900 | 0.734 | 0.496 | |  | | -21.275 | | -0.279 | | | 0.791 | | | |  |
|  |  |  |  | 1991-2000 | |  |  | 106.266 | 0.984 | 0.370 | |  | | 25.709 | | 0.366 | | | 0.729 | | | |  |
|  |  |  |  | 2001-2010 | |  |  | **-242.198** | -3.888 | 0.012 | |  | | -92.415 | | -1.318 | | | 0.245 | | | |  |
|  |  |  |  | 2011-2017 | |  |  | 64.639 | 0.455 | 0.668 | |  | | 125.686 | | 1.254 | | | 0.265 | | | |  |
|  |  | APH Tp (mm) | | 1981-1990 | | 1981-2007 |  | -0.120 | -0.041 | 0.969 | |  | | -1.248 | | -0.621 | | | 0.562 | | | |  |
|  |  |  |  | 1991-2000 | |  |  | 3.506 | 1.405 | 0.219 | |  | | 3.301 | | 1.768 | | | 0.137 | | | |  |
|  |  |  |  | 2001-2007 | |  |  | -6.763 | -2.027 | 0.099 | |  | | -2.932 | | -1.725 | | | 0.145 | | | |  |
|  |  | APH Ap (km^2^) | | 1981-1990 | | 1981-2007 |  | **196.127** | 2.986 | 0.031 | |  | | **232.959** | | 3.453 | | | 0.018 | | | |  |
|  |  |  |  | 1991-2000 | |  |  | -1.464 | -0.017 | 0.987 | |  | | -16.700 | | -0.206 | | | 0.845 | | | |  |
|  |  |  |  | 2001-2007 | |  |  | **-284.676** | -3.277 | 0.022 | |  | | **-308.941** | | | -3.029 | | | | 0.029 | | |

**Extended Table 3. The MK and Sen’s temporal trend analysis along with Sen’s slope estimates for three month running average SSTA during 1981–2017**. Filled cells with bold values represent the significant trends at 95% confidence level. The revised values reflect the influence of serial correlation.

| CO | Atmospheric variables used to define index |  |  | Tarbela | | | |  | | Mangla | | | |  | | Overall | | | |  |
| --- | --- | --- | --- | --- | --- | --- | --- | --- | --- | --- | --- | --- | --- | --- | --- | --- | --- | --- | --- | --- |
|  |  |  |  | | Kendall's tau  (revised tau) | *p*-value  (revised *p*-value) | Original (Bias corrected) Sen's slope  (⁰C three  month^-1^ year^-1^) | |  | | Kendall's tau  (revised tau) | *p*-value  (revised *p*-value) | Sen's slope  (⁰C three month^-1^year-^1^) | |  | | Kendall's tau  (revised tau) | *p*-value  (revised *p*-value) | Original (Bias corrected) Sen's slope  (⁰C three  month^-1^ year^-1^) | |
| ENSO | Three months running based average SSTA | DJF |  | | 0.022(-0.005) | 0.866(0.985) | 0.003(0.003) | |  | | 0.032(0.032) | 0.807(0.837) | 0.006(0.004) | |  | | -0.032(-0.031) | 0.818(0.847) | -0.004(-0.017) | |
|  |  | JFM |  | | 0.035(0.034) | 0.792(0.806) | 0.000(0.000) | |  | | 0.040(0.039) | 0.763(0.797) | 0.005(0.003) | |  | | -0.026(-0.025) | 0.851(0.877) | 0.000(-0.014) | |
|  |  | FMA |  | | 0.013(0.012) | 0.925(0.939) | 0.000(-0.008) | |  | | 0.000(0.000) | 1.000(0.986) | 0.000(-0.011) | |  | | -0.082(-0.079) | 0.558(0.601) | -0.011(-0.017) | |
|  |  | MAM |  | | 0.035(0.034) | 0.792(0.805) | 0.000(0.000) | |  | | 0.003(0.002) | 0.985(1.000) | 0.000(-0.001) | |  | | -0.094(-0.091) | 0.502(0.546) | -0.009(-0.009) | |
|  |  | AMJ |  | | -0.015(-0.015) | 0.910(0.935) | 0.000(-0.008) | |  | | -0.040(-0.039) | 0.763(0.827) | 0.000(-0.005) | |  | | -0.131(-0.128) | 0.346(0.356) | -0.014(-0.007) | |
|  |  | MJJ |  | | -0.030(-0.030) | 0.821(0.791) | 0.000(-0.008) | |  | | -0.040(-0.039) | 0.763(0.777) | 0.000(-0.006) | |  | | -0.084(-0.083) | 0.544(0.558) | -0.010(-0.009) | |
|  |  | JJA |  | | -0.038(-0.037) | 0.778(0.792) | -0.004(-0.011) | |  | | -0.055(-0.054) | 0.679(0.692) | -0.004(-0.007) | |  | | -0.058(-0.057) | 0.676(0.691) | -0.005(-0.009) | |
|  |  | JAS |  | | -0.095(-0.094) | 0.475(0.487) | -0.015(-0.018) | |  | | -0.092(-0.091) | 0.487(0.534) | -0.009(-0.013) | |  | | -0.055(-0.054) | 0.691(0.725) | -0.006(-0.007) | |
|  |  | ASO |  | | -0.080(-0.079) | 0.548(0.560) | -0.010(-0.014) | |  | | -0.062(-0.062) | 0.639(0.679) | -0.007(-0.012) | |  | | 0.003(0.003) | 0.983(1.000) | 0.000(-0.002) | |
|  |  | SON |  | | -0.085(-0.084) | 0.523(0.535) | -0.013(-0.017) | |  | | -0.075(-0.074) | 0.573(0.615) | -0.011(-0.014) | |  | | 0.000(0.000) | 1.000(0.984) | 0.000(-0.001) | |
|  |  | OND |  | | -0.072(-0.071) | 0.586(0.598) | -0.017(-0.019) | |  | | -0.077(-0.076) | 0.560(0.603) | -0.018(-0.019) | |  | | -0.003(-0.003) | 0.983(1.000) | 0.000(-0.007) | |
|  |  | NDJ |  | | -0.070(-0.069) | 0.599(0.611) | -0.020(-0.022) | |  | | -0.067(-0.066) | 0.612(0.625) | -0.019(-0.022) | |  | | -0.075(-0.074) | 0.587(0.602) | -0.020(-0.019) | |
| NAO | Three months running based average SSTA | DJF |  | | -0.094(-0.005) | 0.492(0.985) | -0.008(-0.010) | |  | | -0.054(-0.044) | 0.697(0.804) | -0.005(-0.006) | |  | | -0.100(-0.085) | 0.483(0.544) | -0.010(-0.006) | |
|  |  | JFM |  | | -0.170(-0.155) | 0.195(0.242) | -0.020(-0.014) | |  | | -0.165(-0.155) | 0.209(0.242) | -0.020(-0.024) | |  | | -0.245(-0.239) | 0.073(0.082) | -0.029(-0.036) | |
|  |  | FMA |  | | -0.072(-0.059) | 0.586(0.665) | -0.008(-0.013) | |  | | -0.114(-0.101) | 0.388(0.452) | -0.015(-0.019) | |  | | -0.180(-0.171) | 0.189(0.217) | -0.026(-0.035) | |
|  |  | MAM |  | | -0.044(-0.037) | 0.752(0.792) | -0.004(-0.018) | |  | | -0.094(-0.084) | 0.492(0.420) | -0.011(-0.027) | |  | | -0.077(-0.063) | 0.592(0.659) | -0.008(-0.002) | |
|  |  | AMJ |  | | -0.052(-0.052) | 0.694(0.706) | -0.005(-0.005) | |  | | -0.141(-0.143) | 0.285(0.326) | -0.014(-0.017) | |  | | -0.108(-0.105) | 0.428(0.467) | -0.015(-0.021) | |
|  |  | MJJ |  | | -0.279(-0.273) | 0.034(0.038) | **-0.030(-0.033)** | |  | | -0.264(-0.248) | 0.045(0.040) | **-0.030(-0.037)** | |  | | -0.297(-0.282) | 0.030(0.040) | **-0.037(-0.046)** | |
|  |  | JJA |  | | -0.384(-0.394) | 0.003(0.003) | **-0.048(-0.059)** | |  | | -0.424(-0.416) | 0.001(0.001) | **-0.054(0.068)** | |  | | -0.453(-0.444) | 0.001(0.001) | **-0.069(-0.076)** | |
|  |  | JAS |  | | -0.340(-0.342) | 0.009(0.009) | **-0.048(-0.056)** | |  | | -0.374(-0.372) | 0.004(0.004) | **-0.043(-0.047)** | |  | | -0.390(-0.390) | 0.004(0.004) | **-0.049(-0.059)** | |
|  |  | ASO |  | | -0.143(-0.165) | 0.288(0.275) | -0.018**(-0.025)** | |  | | -0.251(-0.264) | 0.058(0.045) | **-0.031(-0.039)** | |  | | -0.248(-0.251) | 0.073(0.002) | **-0.033(-0.049)** | |
|  |  | SON |  | | 0.039(0.056) | 0.781(0.679) | 0.009(0.003) | |  | | 0.010(0.017) | 0.956(0.910) | 0.001(-0.002) | |  | | 0.026(0.031) | 0.869(0.775) | 0.006(-0.007) | |
|  |  | OND |  | | 0.113(0.113) | 0.402(0.397) | 0.014(0.007) | |  | | 0.079(0.076) | 0.565(0.572) | 0.011(-0.000) | |  | | 0.071(0.063) | 0.621(0.661) | 0.012(0.024) | |
|  |  | NDJ |  | | 0.017(0.027) | 0.896(0.851) | 0.003(-0.004) | |  | | 0.062(0.071) | 0.639(0.506) | 0.007(0.003) | |  | | 0.108(0.117) | 0.428(0.402) | 0.015(0.005) | |
| IOD | Three months running based average SSTA | DJF |  | | 0.118(0.094) | 0.382(0.469) | 0.003(0.000) | |  | | 0.232(0.187) | 0.081(0.143) | 0.005(0.003) | |  | | 0.248(0.225) | 0.073(0.092) | 0.005(0.004) | |
|  |  | JFM |  | | 0.202(0.189) | 0.130(0.058) | 0.006(0.004) | |  | | 0.246(0.236) | 0.063(0.013) | **0.007(0.005)** | |  | | 0.202(0.205) | 0.146(0.071) | 0.007(0.003) | |
|  |  | FMA |  | | 0.167(0.187) | 0.211(0.151) | 0.006(0.005) | |  | | 0.200(0.243) | 0.129(0.007) | **0.009(0.008)** | |  | | 0.143(0.185) | 0.297(0.059) | 0.005(0.007) | |
|  |  | MAM |  | | 0.202(0.209) | 0.130(0.110) | 0.009(0.009) | |  | | 0.217(0.243) | 0.103(0.007) | **0.009(0.012)** | |  | | 0.157(0.182) | 0.264(0.077) | 0.007(0.008) | |
|  |  | AMJ |  | | 0.123(0.140) | 0.362(0.286) | 0.007(0.007) | |  | | 0.182(0.197) | 0.173(0.041) | **0.010(0.013)** | |  | | 0.157(0.159) | 0.264(0.119) | 0.009(0.010) | |
|  |  | MJJ |  | | 0.108(0.115) | 0.424(0.384) | 0.007(0.007) | |  | | 0.212(0.202) | 0.112(0.125) | 0.012(0.013) | |  | | 0.231(0.213) | 0.096(0.119) | 0.012(0.012) | |
|  |  | JJA |  | | 0.138(0.145) | 0.306(0.272) | 0.010(0.007) | |  | | 0.246(0.232) | 0.063(0.08) | 0.019(0.014) | |  | | 0.276(0.267) | 0.045(0.000) | **0.020(0.017)** | |
|  |  | JAS |  | | 0.143(0.101) | 0.288(0.450) | 0.009(0.005) | |  | | 0.232(0.202) | 0.081(0.127) | 0.017(0.017) | |  | | 0.271(0.242) | 0.050(0.004) | **0.021(0.016)** | |
|  |  | ASO |  | | 0.153(0.155) | 0.256(0.242) | 0.013(0.009) | |  | | 0.232(0.239) | 0.081(0.069) | 0.017(0.019) | |  | | 0.282(0.287) | 0.040(0.036) | **0.024(0.025)** | |
|  |  | SON |  | | 0.197(0.187) | 0.140(0.006) | 0.012(0.012) | |  | | 0.266(0.264) | 0.044(0.000) | **0.013(0.017)** | |  | | 0.311(0.305) | 0.024(0.027) | **0.017(0.020)** | |
|  |  | OND |  | | 0.187(0.177) | 0.161(0.179) | 0.010(0.009) | |  | | 0.271(0.266) | 0.040(0.000) | **0.014(0.013)** | |  | | 0.316(0.319) | 0.021(0.026) | **0.015(0.021)** | |
|  |  | NDJ |  | | 0.177(0.189) | 0.185(0.146) | 0.007(0.004) | |  | | 0.296(0.281) | 0.025(0.031) | **0.010(0.009)** | |  | | 0.322(0.307) | 0.019(0.023) | **0.011(0.011)** | |

**Extended Table 4. Same as Extended Table 2 except for MET and AGR seasons (see text for more details)**.

| Climate Oscillation | Atmospheric variables used to define index |  |  | Tarbela | | |  | Mangla | | |  | Overall | | |
| --- | --- | --- | --- | --- | --- | --- | --- | --- | --- | --- | --- | --- | --- | --- |
|  |  |  |  | Kendall's tau  (revised tau) | *p*-value  (revised *p*-value) | Original(Bias corrected)  Sen's slope  (⁰C three month^-1^ year^-1^) |  | Kendall's tau  (revised tau) | *p*-value  (revised *p*-value) | Sen's slope (⁰C season^-1^year^-1^) |  | Kendall's tau  (revised tau) | *p*-value  (revised *p*-value) | Original(Bias corrected)  Sen's slope  (⁰C three month^-1^ year^-1^) |
| ENSO | Three months running average SSTA (MET based) | Pre WD |  | -0.070(-0.069) | 0.599(0.612) | -0.020(-0.022) |  | -0.067(-0.066) | 0.612(0.625) | -0.019(-0.022) |  | -0.075(-0.074) | 0.587(0.602) | -0.020(-0.019) |
|  |  | WD |  | 0.027(0.027) | 0.836(0.851) | 0.005(-0.000) |  | 0.042(0.042) | 0.750(0.784) | 0.008(0.005) |  | -0.023(-0.023) | 0.867(0.893) | -0.013(-0.018) |
|  |  | Post WD |  | -0.020(-0.019) | 0.881(0.895) | -0.001(0.005) |  | 0.000(0.000) | 1.000(0.986) | 0.000(0.008) |  | 0.083(0.083) | 0.545(0.590) | 0.010(0.017) |
|  |  | Pre MS |  | -0.025(-0.024) | 0.851(0.833) | 0.000(-0.013) |  | -0.040(-0.394) | 0.763(0.777) | -0.002(-0.005) |  | -0.113(-0.111) | 0.415(0.427) | -0.008(-0.008) |
|  |  | MS |  | -0.084(-0.084) | 0.523(0.536) | -0.011(-0.016) |  | -0.094(-0.094) | 0.476(0.525) | -0.009(-0.012) |  | -0.060(-0.060) | 0.661(0.629) | -0.005(-0.007) |
|  |  | Post MS |  | -0.091(-0.091) | 0.487(0.499) | -0.018(-0.019) |  | -0.087(-0.086) | 0.511(0.556) | -0.017(-0.015) |  | -0.011(-0.014) | 0.934(0.954) | -0.002(-0.002) |
|  |  |  |  |  |  |  |  |  |  |  |  |  |  |  |
|  | Three months running average SSTA (AGR based) | Pre Rabi |  | -0.077(-0.076) | 0.561(0.573) | -0.017(-0.021) |  | -0.121(-0.121) | 0.358(0.376) | -0.013(-0.017) |  | -0.100(-0.099) | 0.465(0.478) | -0.010(-0.016) |
|  |  | Rabi |  | 0.027(0.027) | 0.836(0.851) | 0.005(-0.000) |  | 0.042(0.042) | 0.750(0.784) | 0.008(0.005) |  | -0.023(-0.023) | 0.867(0.893) | -0.013(-0.018) |
|  |  | Post Rabi |  | 0.020(0.020) | 0.881(0.895) | 0.001(-0.005) |  | 0.000(0.000) | 1.000(0.986) | 0.000(-0.008) |  | -0.083(-0.083) | 0.545(0.591) | -0.010(-0.016) |
|  |  | Pre Kharif |  | -0.025(-0.025) | 0.851(0.833) | 0.000(-0.013) |  | -0.040(-0.039) | 0.763(0.777) | -0.002(-0.005) |  | -0.113(-0.111) | 0.415(0.427) | -0.008(-0.008) |
|  |  | Kharif |  | -0.091(-0.091) | 0.487(0.499) | -0.010(-0.013) |  | -0.109(-0.108) | 0.409(0.458) | -0.008(-0.009) |  | -0.086(-0.085) | 0.531(0.573) | -0.008(-0.009) |
|  |  | Post Kharif |  | -0.091(-0.091) | 0.487(0.499) | -0.018(-0.019) |  | -0.087(-0.086) | 0.511(0.556) | -0.017(-0.014) |  | -0.011(-0.014) | 0.934(0.954) | -0.002(-0.003) |
| NAO | Three months running average SSTA (MET based) | Pre WD |  | 0.017(0.027) | 0.896(0.851) | 0.003(-0.004) |  | 0.062(0.071) | 0.639(0.506) | 0.007(0.003) |  | 0.108(0.117) | 0.428(0.403) | 0.015(0.005) |
|  |  | WD |  | -0.187(-0.209) | 0.161(0.113) | -0.017(-0.012) |  | -0.158(-0.170) | 0.240(0.200) | -0.017(-0.019) |  | -0.236(-0.245) | 0.088(0.075) | -0.031(-0.017) |
|  |  | Post WD |  | -0.094(-0.088) | 0.476(0.509) | -0.009(-0.012) |  | -0.126(-0.125) | 0.339(0.217) | -0.014(-0.021) |  | -0.157(-0.148) | 0.264(0.286) | -0.023(-0.032) |
|  |  | Pre MS |  | -0.187(-0.180) | 0.161(0.175) | -0.019(-0.022) |  | -0.217(-0.207) | 0.103(0.118) | -0.022**(-0.029)** |  | -0.214(-0.211) | 0.124(0.126) | -0.027**(-0.036)** |
|  |  | MS |  | -0.374(-0.374) | 0.004(0.004) | **-0.040(-0.051)** |  | -0.424(-0.416) | 0.001(0.002) | **-0.046(-0.055)** |  | -0.447(-0.439) | 0.001(0.001) | **-0.055(-0.063)** |
|  |  | Post MS |  | 0.094(0.096) | 0.492(0.475) | 0.012(0.007) |  | 0.054(0.059) | 0.697(0.665) | 0.009(-0.004) |  | 0.437(0.484) | 0.805(0.738) | 0.008(-0.003) |
|  |  |  |  |  |  |  |  |  |  |  |  |  |  |  |
|  | Three months running average SSTA (AGR based) | Pre Rabi |  | 0.091(0.096) | 0.488(0.475) | 0.011(-0.000) |  | 0.091(0.094) | 0.488(0.588) | 0.010(-0.000) |  | 0.131(0.125) | 0.337(0.369) | 0.015(0.009) |
|  |  | Rabi |  | -0.187(-0.210) | 0.161(0.113) | -0.017(-0.012) |  | -0.158(-0.170) | 0.240(0.200) | -0.017(-0.019) |  | -0.236(-0.245) | 0.088(0.075) | -0.031(-0.017) |
|  |  | Post Rabi |  | -0.094(-0.088) | 0.476(0.510) | -0.009(-0.015) |  | -0.126(-0.125) | 0.339(0.217) | -0.014(-0.022) |  | -0.157(-0.148) | 0.264(0.286) | -0.023(-0.031) |
|  |  | Pre Kharif |  | -0.187(-0.179) | 0.161(0.175) | -0.019(-0.022) |  | -0.217(-0.207) | 0.103(0.118) | -0.022**(-0.029)** |  | -0.214(-0.211) | 0.124(0.127) | -0.027**(-0.036)** |
|  |  | Kharif |  | -0.404(-0.390) | 0.002(0.003) | **-0.049(-0.057)** |  | -0.432(-0.416) | 0.001(0.002) | **-0.053(-0.059)** |  | -0.462(-0.447) | 0.001(0.001) | **-0.063(-0.074)** |
|  |  | Post Kharif |  | -0.049(-0.049) | 0.724(0.720) | -0.005(-0.015) |  | -0.113(-0.121) | 0.402(0.157) | -0.009(-0.020) |  | -0.088(-0.085) | 0.536(0.543) | -0.009(-0.024) |
| IOD | Three months running average SSTA (MET based) | Pre WD |  | 0.177(0.189) | 0.185(0.146) | 0.007(0.004) |  | 0.296(0.281) | 0.025(0.039) | **0.010(0.009)** |  | 0.322(0.307) | 0.019(0.023) | **0.011(0.012)** |
|  |  | WD |  | 0.172(0.172) | 0.198(0.185) | 0.004(0.003) |  | 0.251(0.239) | 0.058(0.021) | **0.006(0.005)** |  | 0.242(0.207) | 0.080(0.059) | 0.008(0.003) |
|  |  | Post WD |  | 0.187(0.197) | 0.161(0.133) | 0.007(0.007) |  | 0.236(0.229) | 0.075(0.072) | 0.008(0.009) |  | 0.174(0.165) | 0.214(0.095) | 0.006(0.007) |
|  |  | Pre MS |  | 0.128(0.157) | 0.342(0.382) | 0.008(0.006) |  | 0.202(0.185) | 0.130(0.053) | 0.010(0.013) |  | 0.197(0.179) | 0.158(0.073) | 0.009(0.011) |
|  |  | MS |  | 0.148(0.165) | 0.272(0.213) | 0.012(0.009) |  | 0.236(0.251) | 0.075(0.057) | 0.018(0.018) |  | 0.276(0.299) | 0.045(0.000) | **0.023(0.022)** |
|  |  | Post MS |  | 0.207(0.192) | 0.121(0.145) | 0.012(0.008) |  | 0.286(0.281) | 0.030(0.000) | **0.014(0.015)** |  | 0.328(0.322) | 0.017(0.018) | **0.016(0.021)** |
|  |  |  |  |  |  |  |  |  |  |  |  |  |  |  |
|  | Three months running average SSTA (AGR based) | Pre Rabi |  | 0.182(0.177) | 0.173(0.175) | 0.009(0.005) |  | 0.300(0.239) | 0.022(0.000) | **0.011(0.009)** |  | 0.333(0.296) | 0.015(0.029) | **0.013(0.015)** |
|  |  | Rabi |  | 0.172(0.172) | 0.198(0.175) | 0.004(0.003) |  | 0.251(0.239) | 0.058(0.021) | **0.006(0.005)** |  | 0.242(0.208) | 0.080(0.059) | 0.008(0.003) |
|  |  | Post Rabi |  | 0.187(0.197) | 0.161(0.132) | 0.007(0.008) |  | 0.236(0.229) | 0.075(0.021) | **0.008(0.009)** |  | 0.174(0.165) | 0.214(0.095) | 0.006(0.007) |
|  |  | Pre Kharif |  | 0.128(0.115) | 0.342(0.382) | 0.008(0.006) |  | 0.202(0.185) | 0.130(0.053) | 0.010(0.013) |  | 0.197(0.179) | 0.158(0.073) | 0.009(0.012) |
|  |  | Kharif |  | 0.163(0.135) | 0.225(0.308) | 0.010(0.007) |  | 0.256(0.219) | 0.053(0.041) | **0.019(0.013)** |  | 0.293(0.254) | 0.033(0.045) | **0.020(0.016)** |
|  |  | Post Kharif |  | 0.182(0.170) | 0.173(0.022) | **0.013(0.011)** |  | 0.256(0.243) | 0.053(0.000) | **0.016(0.018)** |  | 0.311(0.288) | 0.024(0.040) | **0.020(0.022)** |

**Extended Table 5. Two different classification schemes are used to evaluate the WEs.** Meteorological seasons take into account the two major weather systems responsible for precipitation in the study area. Cropping seasons are to link the WEs and weather systems with low riparian downstream of Tarbela-Indus and Mangla-Jhelum.

| S. No. |  | Meteorological Season | | |  | Cropping Season | |
| --- | --- | --- | --- | --- | --- | --- | --- |
| 1 |  | Pre Western Disturbances | (Pre WD) | Dec |  | Pre Rabi | Nov-Dec |
| 2 |  | Western Disturbances | (WD) | Jan-Feb |  | Rabi* | Jan-Feb |
| 3 |  | Post Western Disturbances | (Post WD) | Mar-Apr |  | Post Rabi | Mar-Apr |
| 4 |  | Pre Monsoon | (Pre MS) | May-Jun |  | Pre Kharif | May-Jun |
| 5 |  | Monsoon | (MS) | Jul-Sep |  | Kharif** | Jul-Aug |
| 6 |  | Post Monsoon | (Post MS) | Oct-Nov |  | Post Kharif | Sep-Oct |
|  |  |  |  |  |  |  |  |

*Rabi (spring) season starts in Sep-Oct and ends in April to June next year depending upon the location. Wheat is the major crop in Pakistan.

** Kharif (summer) season starts in May-Jun and ends in Sep-Oct depending upon the location and crop type. Cotton, Sugarcane, Rice and Maize crops are major irrigated crops.

**Extended Table 6. Landsat 3, 5, 7 and 8 satellite data are used for the extractions of the WEs for Tarbela and Mangla reservoirs**. Temporal frequency cover for Landsat 3 is during 1981–83; for Landsat 5 during 1984–2001 & 2008–2011; for Landsat 7 during 2001–2007 & 2012–2013 and for Landsat 8 during 2013–2017.

| Target Site | Months |  | 1981–1990 | 1991–2000 | 2001–2010 | 2011–2017 |  | Total Images |
| --- | --- | --- | --- | --- | --- | --- | --- | --- |
|  |  |  |  |  |  |  |  |  |
| Landsat grid 150/36 (Tarbela-Indus) | Jan |  | 1 | 4 | 4 | 4 |  | 13 |
|  | Feb |  | 1 | 4 | 0 | 2 |  | 7 |
|  | Mar |  | 1 | 5 | 4 | 3 |  | 13 |
|  | Apr |  | 3 | 4 | 7 | 4 |  | 18 |
|  | May |  | 1 | 9 | 11 | 8 |  | 29 |
|  | Jun |  | 1 | 3 | 4 | 8 |  | 16 |
|  | Jul |  | 0 | 1 | 4 | 1 |  | 6 |
|  | Aug |  | 0 | 4 | 4 | 1 |  | 9 |
|  | Sep |  | 3 | 3 | 6 | 4 |  | 16 |
|  | Oct |  | 1 | 3 | 11 | 8 |  | 23 |
|  | Nov |  | 0 | 5 | 7 | 5 |  | 17 |
|  | Dec |  | 3 | 7 | 4 | 8 |  | 22 |
|  | **Sub-total** |  | **15** | **52** | **66** | **56** |  | **189** |
|  |  |  |  |  |  |  |  |  |
| Landsat grid 149/37 (Mangla-Jhelum) | Jan |  | 1 | 5 | 3 | 4 |  | 13 |
|  | Feb |  | 1 | 7 | 6 | 4 |  | 18 |
|  | Mar |  | 1 | 2 | 6 | 4 |  | 13 |
|  | Apr |  | 0 | 11 | 9 | 9 |  | 29 |
|  | May |  | 3 | 4 | 8 | 7 |  | 22 |
|  | Jun |  | 0 | 2 | 9 | 6 |  | 17 |
|  | Jul |  | 0 | 2 | 4 | 1 |  | 7 |
|  | Aug |  | 0 | 2 | 2 | 4 |  | 8 |
|  | Sep |  | 1 | 5 | 5 | 7 |  | 18 |
|  | Oct |  | 1 | 4 | 9 | 10 |  | 24 |
|  | Nov |  | 0 | 7 | 10 | 10 |  | 27 |
|  | Dec |  | 1 | 2 | 8 | 8 |  | 19 |
|  | **Sub-total** |  | **9** | **53** | **79** | **74** |  | **215** |
|  |  |  |  |  |  |  |  |  |
| **Total** |  |  | **24** | **105** | **145** | **130** |  | **404** |

**Extended Table 7. Same as Extended Fig. 3 except for more detailed information on COs related statistics and floods/drought years recorded in Pakistan.**

| Class | Percentiles |  | ENSO | |  | NAO | |  | IOD | |  | Pakistan | |
| --- | --- | --- | --- | --- | --- | --- | --- | --- | --- | --- | --- | --- | --- |
|  |  |  | Cumulative SSTA  (^o^C) | Years |  | Cumulative SSTA  (^o^C) | Years |  | Cumulative SSTA  (^o^C) | Years |  | Flood Years | Drought Years |
| Extreme Wet | 5 |  | <–9.9 | 1999, 2011 |  | <–6.4 | 1998, 2010 |  | <–1.8 | 1992, 1996 |  | 1983, 1986, 1988, 1989, 1992, 1994, 1995, 2010, 2011, 2014 |  |
| Very Strong | 10 |  | –9.9 to –9.4 | 1988, 2000 |  | –6.4 to –4.5 | 2008, 2012 |  | –1.8 to –1.0 | 1985, 1989 |  |  |  |
| Strong | 20 |  | –9.4 to –6.6 | 1985, 1989, 2007, 2008 |  | –4.5 to -3.4 | 2005, 2006, 2009 |  | –1.0 to 0.1 | 1981, 1984, 1990, 2005 |  |  |  |
| Moderate | 30 |  | –6.6 to – 4.0 | 1984, 1996,2010 |  | –3.4 to -2.7 | 1981, 1985, 1996, 2001 |  | 0.1 to 1.0 | 1986, 1988, 2016 |  |  |  |
| Weak | 40 |  | –4.0 to –2.0 | 1981, 2001, 2013,2017 |  | –2.7 to -1.1 | 1987, 1995, 1997, 2016 |  | 1.0 to 1.4 | 1983, 1993, 1998, 2004 |  |  |  |
| Neutral | 50 |  | –2.0 to 0.4 | 1995, 1998, 2012 |  | –1.1 to 1.1 | 1988, 2002, 2003, 2014 |  | 1.4 to 1.8 | 1995, 2001, 2013, 2014 |  |  |  |
| Weak | 60 |  | 0.4 to 3.0 | 1986, 2005, 2006, 2014 |  | 1.1 to 1.9 | 1993, 2004, 2008, 2013 |  | 1.8 to 2.6 | 1999, 2002, 2003, 2010 |  |  | 1982, 1987,  1998-2002,  2004-2005,  2009-2010 |
| Moderate | 70 |  | 3.0 to 4.0 | 1990, 1993, 2003, 2009 |  | 1.9 to 2.5 | 2000, 2011, 2017 |  | 2.6 to 3.8 | 2000, 2008, 2009 |  |  |  |
| Strong | 80 |  | 4.0 to 5.7 | 1983, 1994, 2004, 2016 |  | 2.5 to 3.9 | 1983, 1984, 1991 |  | 3.8 to 4.3 | 1987, 1991, 2006, 2007 |  |  |  |
| Very Strong | 90 |  | 5.7 to 9.5 | 1991, 1992, 2002 |  | 3.9 to 5.7 | 1982, 1986, 1999, 2015 |  | 4.3 to 5.4 | 2011, 2012, 2015 |  |  |  |
| Extreme Dry | 100 |  | >9.5 | 1982, 1987, 1997, 2015 |  | >5.7 | 1989, 1990, 1992, 1994 |  | >5.4 | 1982, 1994, 1997, 2017 |  |  |  |
|  |  |  |  |  |  |  |  |  |  |  |  |  |  |

Red and blue colors show the drought and flood years respectively, and are linked to associated SSTA classes.

**Extended Table 8. List of variables used in development of the multiple linear regression models for Tarbela and Mangla reservoirs.** See text for more details.

| Reservoirs | Total Variables | Year and CO Indices |  | ERA | | |  | APH | | |
| --- | --- | --- | --- | --- | --- | --- | --- | --- | --- | --- |
|  |  |  |  | Total Precipitation | Precipitation  Area | Glaciated Total and Area Precipitation |  | Total Precipitation | Precipitation  Area | Glaciated Total and Area Precipitation |
| Tarbela | 148 | Years (1981-2017); ENSO SSTA; NAO SSTA; IOD SSTA |  | ERA Tp; ERA Tp T1; ERA Tp T2; ERA Tp T3; ERA Tp T4; ERA Tp T5; ERA Tp T6; ERA Tp T7; ERA Tp T8; ERA Tp T9; ERA Tp T10; ERA Tp T11; ERA Tp T12; ERA Tp T13; ERA Tp T14; ERA Tp T15; ERA Tp T16; ERA Tp T17; ERA Tp T18; ERA Tp T19; ERA Tp T20; ERA Tp T21; ERA Tp T22; ERA Tp T23; ERA Tp T24; ERA Tp T25; ERA Tp T26 | ERA Ap; ERA Ap T1; ERA Ap T2; ERA Ap T3; ERA Ap T4; ERA Ap T5; ERA Ap T6; ERA Ap T7; ERA Ap T8; ERA Ap T9; ERA Ap T10; ERA Ap T11; ERA Ap T12; ERA Ap T13; ERA Ap T14; ERA Ap T15; ERA Ap T16; ERA Ap T17; ERA Ap T18; ERA Ap T19; ERA Ap T20; ERA Ap T21; ERA Ap T22; ERA Ap T23; ERA Ap T24; ERA Ap T25; ERA Ap T26 | ERA Tp GG20; ERA Tp GB520; ERA Tp GL5; ERA Tp GG20P; ERA Tp GB520P; ERA Tp GL5P; ERA Tp GG20D; ERA Tp GB520D; ERA Tp GL5D  ERA Ap GG20; ERA Ap GB520; ERA Ap GL5; ERA Ap GG20P; ERA Ap GB520P; ERA Ap GL5P; ERA Ap GG20D; ERA Ap GB520D; ERA Ap GL5D |  | APH Tp; APH Tp T1; APH Tp T2; APH Tp T3; APH Tp T4; APH Tp T5; APH Tp T6; APH Tp T7; APH Tp T8; APH Tp T9; APH Tp T10; APH Tp T11; APH Tp T12; APH Tp T13; APH Tp T14; APH Tp T15; APH Tp T16; APH Tp T17; APH Tp T18; APH Tp T19; APH Tp T20; APH Tp T21; APH Tp T22; APH Tp T23; APH Tp T24; APH Tp T25; APH Tp T26 | APH Ap; APH Ap T1; APH Ap T2; APH Ap T3; APH Ap T4; APH Ap T5; APH Ap T6; APH Ap T7; APH Ap T8; APH Ap T9; APH Ap T10; APH Ap T11; APH Ap T12; APH Ap T13; APH Ap T14; APH Ap T15; APH Ap T16; APH Ap T17; APH Ap T18; APH Ap T19; APH Ap T20; APH Ap T21; APH Ap T22; APH Ap T23; APH Ap T24; APH Ap T25; APH Ap T26 | APH Tp GG20; APH Tp GB520; APH Tp GL5; APH Tp GG20P; APH Tp GB520P; APH Tp GL5P; APH Tp GG20D; APH Tp GB520D; APH Tp GL5D  APH Ap GG20; APH Ap GB520; APH Ap GL5; APH Ap GG20P; APH Ap GB520P; APH Ap GL5P; APH Ap GG20D; APH Ap GB520D and APH Ap GL5D |
| Mangla | 40 | Years (1981-2017); ENSO SSTA; NAO SSTA; IOD SSTA |  | ERA Tp; ERA Tp M1; ERA Tp M2; ERA Tp M3; ERA Tp M4; ERA Tp M5 | ERA Ap; ERA Ap M1; ERA Ap M2; ERA Ap M3; ERA Ap M4; ERA Ap M5 | ERA Tp GL5; ERA Tp GL5P; ERA Tp GL5D  ERA Ap GL5; ERA Ap GL5P; ERA Ap GL5D |  | APH Tp; APH Tp M1; APH Tp M2; APH Tp M3; APH Tp M4; APH Tp M5 | APH Ap; APH Ap M1; APH Ap M2; APH Ap M3; APH Ap M4; APH Ap M5 | APH Tp GL5; APH Tp GL5P; APH Tp GL5D  APH Ap GL5; APH Ap GL5P and APH Ap GL5D |


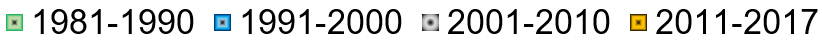


**Tarbela**

**Mangla**

Decadal Change (%)

**Extended Fig. 1. Same as Extended Table 2 except for percent decadal changes.** Analysis shows an alarming decrease in WEs for both reservoirs.

NAO

IOD

ENSO

95% confidence level

**Extended Fig. 2. Relationship of Tp, Ap with ENSO, NAO and IOD for Tarbela, Mangla and overall basin based on monthly correlation coefficients.** The ENSO shows more strong and significant correlations with Tp and Ap as compared to NAO and IOD.

**Extended Fig. 3. Annual COs classification scheme based on percentiles applied to cumulative SSTA for ENSO, NAO and IOD**. This scheme has identified extreme dry and wet years and is used to link flood and drought events across south Asia including Pakistan. Flood events occurrence is increasing significantly since 1981. Extreme wet/dry conditions have been observed for year 1999/2005 (ENSO), 2010/1989 (NAO) and 1992/1997 (IOD).

**Extended Fig. 4. An integrated approach is developed to assess the co-variability between satellites data-based WE with basin wide precipitation and COs.** First step is the extraction of the reservoir’s specific watershed basin from ALOS GDEM and refinements. Two identified Landsat grids covering the reservoirs are downloaded with no cloud cover present over the water reservoir. The NDWI based WE is linked with ERA and APH Tp and Ap along with ENSO, NAO and IOD based SSTA to explain the fact behind the current decreasing trend in water storages. The statistical techniques of linear trends, correlation coefficients, MK trends, percent changes and multiple regression models are employed to quantify the relationships.
